# Return to the Sea, Get Huge, Beat Cancer: An Analysis of Cetacean Genomes Including an Assembly for the Humpback Whale (*Megaptera novaeangliae*)

**DOI:** 10.1093/molbev/msz099

**Published:** 2019-05-09

**Authors:** Marc Tollis, Jooke Robbins, Andrew E Webb, Lukas F K Kuderna, Aleah F Caulin, Jacinda D Garcia, Martine Bèrubè, Nader Pourmand, Tomas Marques-Bonet, Mary J O’Connell, Per J Palsbøll, Carlo C Maley

**Affiliations:** 1 Biodesign Institute, Arizona State University, Tempe, AZ; 2 School of Life Sciences, Arizona State University, Tempe, AZ; 3 School of Informatics, Computing, and Cyber Systems, Northern Arizona University, Flagstaff, AZ; 4 Center for Coastal Studies, Provincetown, MA; 5 Center for Computational Genetics and Genomics, Temple University, Philadelphia, PA; 6 Instituto de Biologia Evolutiva (UPF-CSIC), PRBB, Barcelona, Spain; 7 Genomics and Computational Biology Program, University of Pennsylvania, Philadelphia, PA; 8 Groningen Institute of Evolutionary Life Sciences, University of Groningen, Groningen, The Netherlands; 9 Jack Baskin School of Engineering, University of California Santa Cruz, Santa Cruz, CA; 10 CNAG‐CRG, Centre for Genomic Regulation (CRG), The Barcelona Institute of Science and Technology, Barcelona, Spain; 11 Institució Catalana de Recerca i Estudis Avançats (ICREA), Barcelona, Catalonia, Spain; 12 Institut Català de Paleontologia Miquel Crusafont, Universitat Autònoma de Barcelona, Edifici ICTA-ICP, Barcelona, Spain; 13 Computational and Molecular Evolutionary Biology Research Group, School of Life Sciences, University of Nottingham, Nottingham, United Kingdom

**Keywords:** cetaceans, humpback whale, evolution, genome, cancer

## Abstract

Cetaceans are a clade of highly specialized aquatic mammals that include the largest animals that have ever lived. The largest whales can have ∼1,000× more cells than a human, with long lifespans, leaving them theoretically susceptible to cancer. However, large-bodied and long-lived animals do not suffer higher risks of cancer mortality than humans—an observation known as Peto’s Paradox. To investigate the genomic bases of gigantism and other cetacean adaptations, we generated a de novo genome assembly for the humpback whale (*Megaptera novaeangliae*) and incorporated the genomes of ten cetacean species in a comparative analysis. We found further evidence that rorquals (family Balaenopteridae) radiated during the Miocene or earlier, and inferred that perturbations in abundance and/or the interocean connectivity of North Atlantic humpback whale populations likely occurred throughout the Pleistocene. Our comparative genomic results suggest that the evolution of cetacean gigantism was accompanied by strong selection on pathways that are directly linked to cancer. Large segmental duplications in whale genomes contained genes controlling the apoptotic pathway, and genes inferred to be under accelerated evolution and positive selection in cetaceans were enriched for biological processes such as cell cycle checkpoint, cell signaling, and proliferation. We also inferred positive selection on genes controlling the mammalian appendicular and cranial skeletal elements in the cetacean lineage, which are relevant to extensive anatomical changes during cetacean evolution. Genomic analyses shed light on the molecular mechanisms underlying cetacean traits, including gigantism, and will contribute to the development of future targets for human cancer therapies.

## Introduction

Cetaceans (whales, dolphins, and porpoises) are highly specialized mammals adapted to an aquatic lifestyle. Diverging from land-dwelling artiodactyls during the late Paleocene or early Eocene ∼55 Ma (Thewissen et al. 2007; O’Leary and Gatesy 2008), cetaceans diversified throughout the Cenozoic and include two extant groups: Mysticeti or the baleen whales, and Odontoceti or the toothed whales. Traits evolved for life in the ocean, including the loss of hind limbs, changes in skull morphology, physiological adaptations for deep diving, and underwater acoustic abilities including echolocation make these species among the most diverged mammals from the ancestral eutherian (Berta et al. 2015). One striking aspect of cetacean evolution is the large body sizes achieved by some lineages, rivaled only by the gigantic terrestrial sauropod dinosaurs (Benson et al. 2014). Cetaceans were not limited by gravity in the buoyant marine environment and evolved multiple giant forms, exemplified today by the largest animal that has ever lived: the blue whale (*Balaenoptera musculus*). Based on evidence from fossils, molecules, and historical climate data, it has been hypothesized that oceanic upwelling during the Pliocene–Pleistocene supported the suspension feeding typical of modern baleen whales, allowing them to reach their gigantic sizes surprisingly close to the present time (Slater et al. 2017).

Although the largest whales arose relatively recently, large body size has evolved multiple times throughout the history of life (Heim et al. 2015), including in 10 out of 11 mammalian orders (Baker et al. 2015). Animal gigantism is therefore a recurring phenomenon that is seemingly governed by available resources and natural selection (Vermeij 2016), where positive fitness consequences lead to repeated directional selection toward larger bodies within populations (Kingsolver and Pfennig 2004). However, there are tradeoffs associated with large body size, including a higher lifetime risk of cancer due to a greater number of somatic cell divisions over time (Peto et al. 1975; Nunney 2018). Surprisingly, although cancer should be a body mass- and age-related disease, large and long-lived animals do not suffer higher cancer mortality rates than smaller, shorter-lived animals (Abegglen et al. 2015). This is a phenomenon known as Peto’s Paradox (Peto et al. 1975). To the extent that there has been selection for large body size, there likely has also been selection for cancer suppression mechanisms that allow an organism to grow large and successfully reproduce. Recent efforts have sought to understand the genomic mechanisms responsible for cancer suppression in gigantic species (Abegglen et al. 2015; Caulin et al. 2015; Keane et al. 2015; Sulak et al. 2016). An enhanced DNA damage response in elephant cells has been attributed to ∼20 duplications of the tumor suppressor gene *TP53* in elephant genomes (Abegglen et al. 2015; Sulak et al. 2016). The bowhead whale (*Balaena mysticetus*) is a large whale that may live more than 200 years (George et al. 1999), and its genome shows evidence of positive selection in many cancer- and aging-associated genes including ERCC1, which is part of the DNA repair pathway (Keane et al. 2015). Additionally, the bowhead whale genome contains duplications of the DNA repair gene *PCNA*, as well as *LAMTOR1*, which helps control cellular growth (Keane et al. 2015). Altogether, these results suggest that 1) the genomes of larger and longer-lived mammals may hold the key to multiple mechanisms for suppressing cancer, and 2) as the largest animals on Earth, whales make very promising sources of insight for cancer suppression research.

Cetacean comparative genomics is a rapidly growing field, with 13 complete genome assemblies available on NCBI as of late 2018, including the following that were available at the onset of this study: the common minke whale (*Balaenoptera acutorostrata*) (Yim et al. 2014), bottlenose dolphin (*Tursiops truncatus*), orca (*Orcinus orca*) (Foote et al. 2015), and sperm whale (*Physeter macrocephalus*) (Warren et al. 2017). In addition, the Bowhead Whale Genome Resource has supported the genome assembly for that species since 2015 (Keane et al. 2015). However, to date, few studies have used multiple cetacean genomes to address questions about genetic changes that have controlled adaptations during cetacean evolution, including the evolution of cancer suppression. Here, we provide a comparative analysis that is novel in scope, leveraging whole-genome data from ten cetacean species, including six cetacean genome assemblies, and a de novo genome assembly for the humpback whale (*Megaptera novaeangliae*). Humpback whales are members of the family Balaenopteridae (rorquals) and share a recent evolutionary history with other ocean giants such as the blue whale and fin whale (*Balaenoptera physalus*) (Árnason et al. 2018). They have an average adult length of more than 13 m (Clapham and Mead 1999), and a lifespan that may extend to 95 years (Chittleborough 1959; Gabriele et al. 2010), making the species an excellent model for Peto’s Paradox research.

Our goals in this study were 3-fold: 1) to provide a de novo genome assembly and annotation for the humpback whale that will be useful to the cetacean research and mammalian comparative genomics communities; 2) to leverage the genomic resource and investigate the molecular evolution of cetaceans in terms of their population demographics, phylogenetic relationships and species divergence times, and the genomics underlying cetacean-specific adaptations; and 3) to determine how selective pressure variation on genes involved with cell cycle control, cell signaling and proliferation, and many other pathways relevant to cancer may have contributed to the evolution of cetacean gigantism. The latter has the potential to generate research avenues for improving human cancer prevention, and perhaps even therapies.

## Results and Discussion

### Sequencing, Assembly, and Annotation of the Humpback Whale Genome

We sequenced and assembled a reference genome for the humpback whale using high-coverage paired-end and mate-pair libraries (table 1, NCBI BioProject PRJNA509641) and obtained an initial assembly that was 2.27 Gb in length, with 24,319 scaffolds, a contig N50 length of 12.5 kb and a scaffold N50 length of 198 kb. Final sequence coverage for the initial assembly was ∼76×, assuming an estimated genome size of 2.74 Gb from a 27-mer spectrum analysis. Hi Rise scaffolding using proximity ligation (Chicago) libraries (Putnam et al. 2016, table 1, NCBI BioProject PRJNA509641) resulted in a final sequence coverage of ∼102×, greatly improving the contiguity of the assembly by reducing the number of scaffolds to 2,558 and increasing the scaffold N50 length 46-fold to 9.14 Mb (table 2). The discrepancy between estimated genome size and assembly length has been observed in other cetacean genome efforts (Keane et al. 2015), and is likely due to the highly repetitive nature of cetacean genomes (Árnason and Widegren 1989).

**Table 1.** Genomic Sequence Data Obtained for the Humpback Whale Genome.

Libraries	Est. Number of Reads	Avg. Read Length (bp)	Est. Depth (total)
180 bp paired-end	1,211,320,000	94	41.3
300 bp paired-end	25,820,000	123	1.2
500 bp paired-end	112,400,000	123	5.0
600 bp paired-end	395,500,000	93	13.4
2 kb mate-paired	348,080,000	49	6.2
10 kb mate-paired	279,000,000	94	9.0
Subtotal for WGS libraries	2,372,120,000		76.1
Chicago Library 1	72,000,000	100	5.3
Chicago Library 2	6,000,000	151	0.7
Chicago Library 3	190,000,000	100	13.9
Chicago Library 4	79,000,000	100	5.8
Subtotal for Chicago Libraries	347,000,000		25.6
Total for all sequence libraries	2,719,120,000		101.7

Note.—WGS, whole-genome shotgun.

**Table 2.** Statistics for the Humpback Whale Genome Assembly.

Feature	Initial Assembly	Final Assembly
Assembly length	2.27 Gb	2.27 Gb
Contig N50	12.4 kb	12.3 kb
Longest scaffold	2.2 Mb	29.4 Mb
Number of scaffolds	24,319	2,558
Scaffold N50	198 kb	9.14 Mb
Scaffold N90	53 kb	2.35 Mb
Scaffold L50	3,214	79
Scaffold L90	11,681	273
Percent genome in gaps	5.36%	5.45%
BUSCO^a^ results—vertebrata	C: 85%[D: 1.8%], F: 15%, M: 4.9%, *n*: 3,023	
BUSCO^a^ results—laurasiatheria	C: 91.2%[D: 0.8%], F: 4.8%, M: 4.0%, *n*: 6,253	
CEGMA^a^ results	C: 226 (91.13%), P: 240 (96.77%)	

Note.—BUSCO, Benchmarking Universal Single Copy Orthologs (Simão et al. 2015): C, complete; D, duplicated; F, fragmented; M, missing. CEGMA, Core Eukaryotic Genes Mapping Approach (Parra et al. 2009): C, complete; P, complete and/or partial.

a BUSCO and CEGMA results for final assembly only.

With 95–96% of near-universal orthologs from OrthoDB v9 (Simão et al. 2015) present in the assembly, as well as 97% of a set of core eukaryotic genes (Parra et al. 2009), the estimated gene content of the humpback whale genome assembly suggests a high-quality genome with good gene representation (table 1). To aid in genome annotation, we carried out skin transcriptome sequencing, which resulted in 281,642,354 reads (NCBI BioProject PRJNA509641). These were assembled into a transcriptome that includes 67% of both vertebrate and laurasiatherian orthologs, and we predicted 10,167 protein-coding genes with likely ORFs that contain BLAST homology to SwissProt proteins (UniProt Consortium 2015). The large number of missing genes from the transcriptome may be due to the small proportion of genes expressed in skin. Therefore, we also assessed homology with ten mammalian proteomes from NCBI and the entire SwissProt database, and ab initio gene predictors (see Materials and Methods, supplementary Methods, and supplementary fig. 1, Supplementary Material online) for gene-calling. The final genome annotation resulted in 24,140 protein-coding genes, including 5,446 with 5′-untranslated regions (UTRs) and 6,863 with 3′-UTRs. We detected 15,465 one-to-one orthologs shared with human and 14,718 with cow. When we compared gene annotations across a sample of mammalian genomes, the humpback whale and bottlenose dolphin genome assemblies had on average significantly shorter introns (*P* = 0.04, unpaired *T*-test, supplementary table 1, Supplementary Material online), which may in part explain the smaller genome size of cetaceans compared with most other mammals (Zhang and Edwards 2012).

We estimated that between ∼30% and ∼39% of the humpback whale genome comprised repetitive elements (table 3). Masking the assembly with a library of known mammalian elements resulted in the identification of more repeats than a de novo method, suggesting that clade-specific repeat libraries are highly valuable when assessing repetitive content. The most abundant group of transposable elements in the humpback whale genome was the autonomous non-long terminal repeat (LTR) retrotransposons (long interspersed nuclear elements or LINEs), which comprised nearly 20% of the genome, most of which belong to the LINE-1 clade as is typical of placental mammals (Boissinot and Sookdeo 2016). Large numbers of nonautonomous non-LTR retrotransposons in the form of short interspersed nuclear elements (SINEs) were also detected; in particular, over 3% of the genome belonged to mammalian inverted repeats. Although the divergence profile of de novo-derived repeat annotations in humpback whale included a decreased average genetic distance within transposable element subfamilies compared with the database-derived repeat landscape, both repeat libraries displayed a spike in the numbers of LINE-1 and SINE retrotransposon subfamilies near 5% divergence, as did the repeat landscapes of the bowhead whale, orca and dolphin, suggesting recent retrotransposon activity in cetaceans (supplementary figs. 2 and 3, Supplementary Material online).

**Table 3.** Repetitive Content of the Humpback Whale (*Megaptera novaeangliae*) Genome, Estimated with a Library of Known Mammalian Repeats (RepBase) and De Novo Repeat Identification (RepeatModeler).

	RepBase		RepeatModeler	
Repeat Type	Length (bp)	% Genome (38.85 total)	Length (bp)	% Genome (30.25 total)
SINEs	137,574,621	6.07	75,509,694	3.33
LINEs	440,955,223	19.46	432,017,456	19.07
LTR	142,117,286	6.27	94,177,184	4.16
DNA transposons	84,243,186	3.72	54,015,996	2.38
Unclassified	1,303,231	0.06	4,339,463	0.19
Satellites	48,894,580	2.16	197,862	0.01
Simple repeats	20,779,839	0.92	20,848,394	0.92
Low complexity	4,167,187	0.18	4,281,173	0.19

### Slow DNA Substitution Rates in Cetaceans and the Divergence of Modern Whale Lineages

**Fig. 1.**
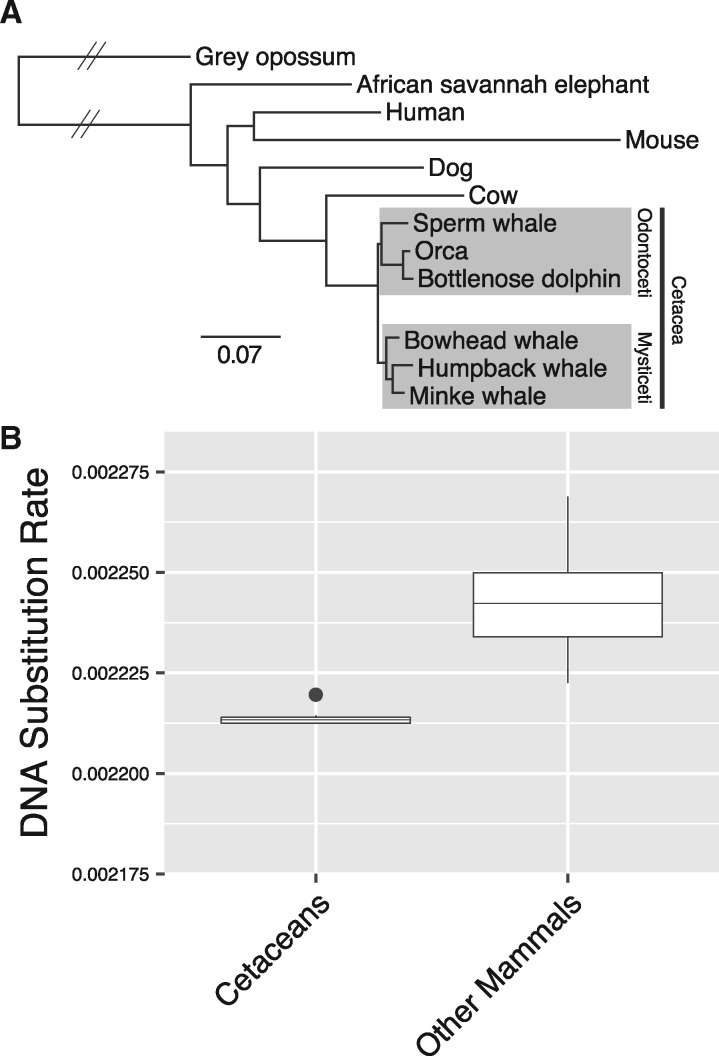
Substitution rates in cetacean genomes. (*A*) Maximum likelihood phylogeny of 12 mammals based on 2,763,828 fourfold degenerate sites. Branch lengths are given in terms of substitutions per site, except for branches with hatched lines which are shortened for visual convenience. All branches received 100% bootstrap support. (*B*) Based on the phylogeny in *(A)*, a comparison of the estimated DNA substitution rates (in terms of substitutions per site per million years) between terminal and internal cetacean branches, and terminal and internal branches of all other mammals.

We computed a whole-genome alignment (WGA) of 12 mammals including opossum, elephant, human, mouse, dog, cow, sperm whale, bottlenose dolphin, orca, bowhead whale, common minke whale, and humpback whale (supplementary table 2, Supplementary Material online), and employed human gene annotations to extract 2,763,828 homologous 4-fold degenerate (4D) sites. A phylogenetic analysis of the 4D sites yielded the recognized evolutionary relationships (fig. 1*A*), including reciprocally monophyletic Mysticeti and Odontoceti. When we compared the substitutions per site along the branches of the phylogeny, we found a larger number of substitutions along the mouse (rodent) branch, supporting negative relationships between generation time (Wilson Sayres et al. 2011), speciation (Pagel et al. 2006), and substitution rates. When applying a semiparametric penalized likelihood (PL) method to estimate substitution rate variation at 4D sites across the 12 mammals, we found that cetaceans have accumulated the lowest number of DNA substitutions per site per million years (fig. 1*B* and supplementary table 3, Supplementary Material online), which may be attributed to long generation times or slower mutation rates in cetaceans (Jackson et al. 2009). Germline mutation rates are related to somatic mutation rates within species (Milholland et al. 2017); therefore, it is possible that slow mutation rates may limit neoplastic progression and contribute to cancer suppression in cetaceans, which is a prediction of Peto’s Paradox (Caulin and Maley 2011).

We also obtained 152 single-copy orthologs (single-gene ortholog families or SGOs, see Materials and Methods and supplementary Methods, Supplementary Material online) identified in at least 24 out of 28 species totaling 314,844 bp, and reconstructed gene trees that were binned and analyzed using a species tree method that incorporates incomplete lineage sorting (see Materials and Methods, Zhang et al. 2018). The species tree topology (supplementary fig. 4, Supplementary Material online) also included full support for the accepted phylogenetic relationships within Cetacea, as well as within Mysticeti and Odontoceti. Lower local posterior probabilities for two of the internal branches within laurasiatherian mammals were likely due to the extensive gene tree heterogeneity that has complicated phylogenetic reconstruction of the placental mammalian lineages (Tarver et al. 2016).

**Fig. 2.**
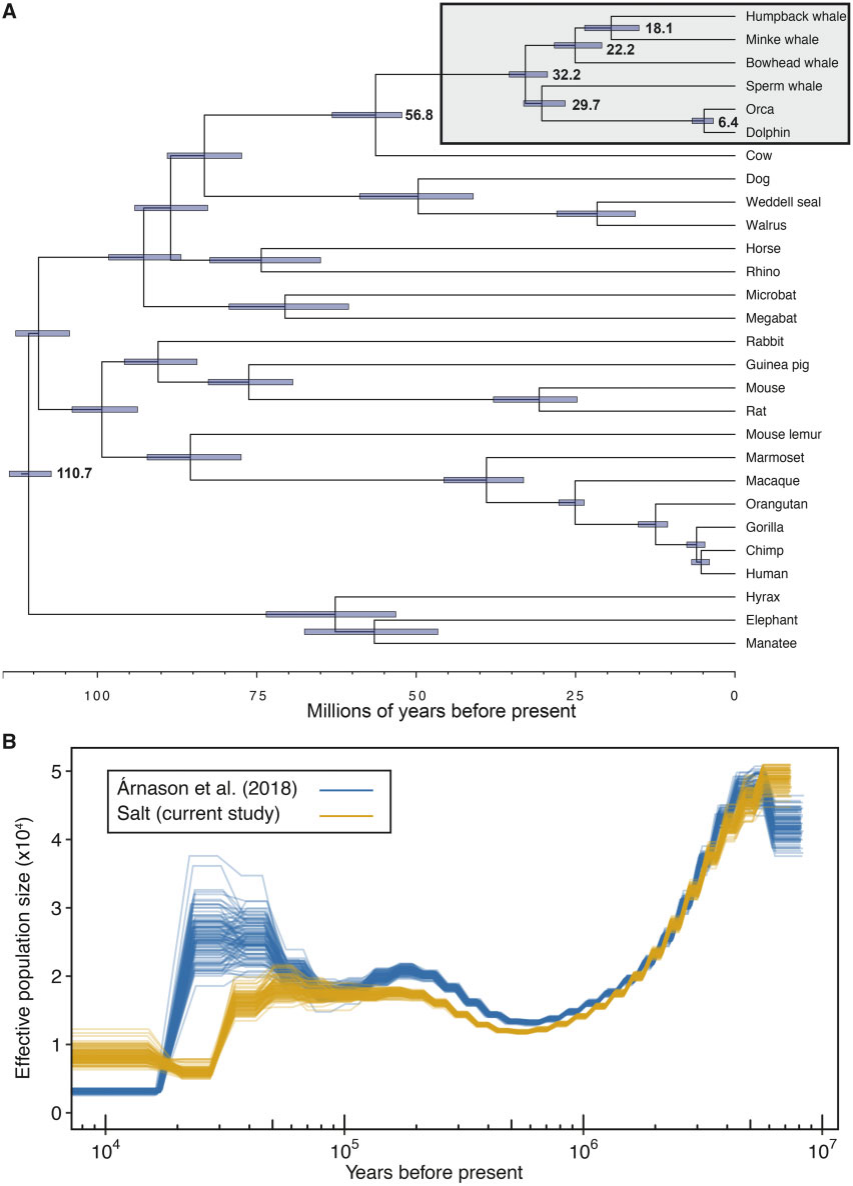
Timescale of humpback whale evolution. (*A*) Species phylogeny of 28 mammals constructed from 152 orthologs and time-calibrated using MCMCtree. Branch lengths are in terms of millions of years. Node bars indicate 95% highest posterior densities of divergence times. Cetaceans are highlighted in the gray box with mean estimates of divergence times included. (*B*) The effective population size (*N*_e_) changes over time. Demographic histories of two North Atlantic humpback whales estimated from the PSMC analysis, including 100 bootstrap replicates per analysis. Mutation rate used was 1.54e-9 per year and generation time used was 21.5 years.

We estimated divergence times in a Bayesian framework using the 4D and SGO data sets independently in MCMCtree (Yang and Rannala 2006), resulting in similar posterior distributions and parameter estimates, with overlapping highest posterior densities for the estimated divergence times of shared nodes across the 4D and SGO phylogenies (supplementary figs. 5 and 6 and tables 4 and 5, Supplementary Material online). We estimated that the time to the most recent common ancestor (TMRCA) of placental mammals was 100–114 Ma during the late Cretaceous, the TMRCA of cow and cetaceans (Cetartiodactyla) was 52–65 Ma during the Eocene or Paleocene, the TMRCA of extant cetaceans was 29–35 Ma during the early Oligocene or late Eocene (between the two data sets), the TMRCA of baleen whales was placed 9–26 Ma in the early Miocene or middle Oligocene, and the TMRCA of humpback and common minke whales (family Balaenopteridae) was 4–22 Ma during the early Pliocene or the Miocene (fig. 2*A*).

### A Complex Demographic History of North Atlantic Humpback Whales

We estimated the demographic history of the North Atlantic humpback whale population applying the Pairwise Sequential Markovian Coalescent (PSMC) (Li and Durbin 2011) to the short-insert libraries generated during this study, as well as sequence reads from a second North Atlantic humpback whale (Árnason et al. 2018) (fig. 2*B*, supplementary figs. 7 and 8, Supplementary Material online). Consistent with the findings of Árnason et al. (2018), we estimated that the largest humpback whale population sizes were ≥2 Ma during the Pliocene–Pleistocene transition, followed by a steady decline until ∼1 Ma. The PSMC trajectories of the two humpback whales began to diverge ∼100,000 years ago, and the estimated confidence intervals from 100 bootstraps for each PSMC analysis were nonoverlapping in the more recent bins. Both humpback PSMC trajectories suggested sharp population declines beginning ∼25,000–45,000 years ago. However, interpreting inferred PSMC plots of past “demographic” changes is nontrivial in a globally distributed species connected by repeated, occasional gene flow such as humpback whales (Baker et al. 1993; Palsbøll et al. 1995; Jackson et al. 2014). The apparent changes in effective population size may represent changes in abundance, interocean connectivity or a combination of both (Hudson 1990; Palsbøll et al. 2013). Several genetic and genome-based studies of cetaceans have demonstrated how past large-scale oceanic changes have affected the evolution of cetaceans (Steeman et al. 2009), including baleen whales (Árnason et al. 2018). Although the population genetic structure of humpback whales in the North Atlantic is not fully resolved, the level of genetic divergence among areas is very low (Larsen et al. 1996; Valsecchi et al. 1997). Therefore, the difference between the two humpback whale PSMC trajectories may be due to recent admixture (Baker et al. 1993; Palsbøll et al. 1995; Ruegg et al. 2013; Jackson et al. 2014), intraspecific variation and population structure (Mazet et al. 2016), as well as errors due to differences in sequence coverage (Nadachowska-Brzyska et al. 2016).

### Segmental Duplications in Cetacean Genomes Contain Genes Involved in Apoptosis and Tumor Suppression

Mammalian genomes contain gene-rich segmental duplications (Alkan et al. 2009), which may represent a powerful mechanism by which new biological functions can arise (Kaessmann 2010). We employed a read-mapping approach to annotate large segmental duplications (LSDs) ≥10 kb in the humpback whale genome assembly and ten additional cetaceans for which whole-genome shotgun data were available (see Materials and Methods, supplementary Methods, and supplementary table 6, Supplementary Material online). We found that cetacean genomes contained on average 318 LSDs (±56 SD), which comprised ∼9.9 Mb (±1.8 Mb) and averaged ∼31 kb in length (±2.4 kb). We identified 10,128,534 bp (0.4%) of the humpback whale genome assembly that comprised 293 LSDs averaging 34,568 bp in length. Fifty-one of the LSDs were shared across all 11 cetacean genomes (supplementary fig. 9, Supplementary Material online). In order to determine the potential role of segmental duplications during the evolution of cetacean-specific phenotypes, we identified 426 gene annotations that overlapped cetacean LSDs, including several genes annotated for viral response. Other genes on cetacean LSDs were involved in aging, in particular *DLD* in the bowhead whale and *KCNMB1* in the blue whale; this may reflect relevant adaptations contributing to longevity in two of the largest and longest-lived mammals (Ohsumi 1979; George et al. 1999). Multiple tumor suppressor genes were located on cetacean LSDs, including 1) *SALL4* in the sei whale; 2) *TGM3* and *SEMA3B* in the orca; 3) *UVRAG* in the sperm whale, North Atlantic right whale, and bowhead whale; and 4) *PDCD5*, which is upregulated during apoptosis (Zhao et al. 2015) and was found in LSDs of all 11 queried cetacean genomes. *PDCD5* pseudogenes have been identified in the human genome, and several Ensembl-hosted mammalian genomes contain one-to-many *PDCD5* orthologs; however, we annotated only a single copy of *PDCD5* in the humpback whale assembly. This suggests that in many cases, gene duplications are collapsed during reference assembly but can be retrieved through shotgun read-mapping methods (Carbone et al. 2014). We annotated fully resolved *SALL4* and *UVRAG* copy number variants in the humpback whale genome assembly, and by mapping the RNA-Seq data from skin to the genome assembly and annotation (see Materials and methods), we found that three annotated copies of *SALL4* were expressed in humpback whale skin, as were two copies of *UVRAG*.

We also found that ∼1.45 Mb (±923 kb) of each cetacean genome consists of LSDs not found in other cetaceans, making them species-specific, which averaged ∼24.4 kb (±14.6 kb) in length (supplementary table 7 and fig. 9, Supplementary Material online). The minke whale genome contained the highest number of genes on its species-specific LSDs (32). After merging the LSD annotations for the two humpback whales, we identified 57 species-specific LSDs for this species, comprising ∼977 kb and containing nine duplicated genes. Humpback whale-specific duplications included the genes *PRMT2*, which is involved in growth and regulation and promotes apoptosis, *SLC25A6* which may be responsible for the release of mitochondrial products that trigger apoptosis, and *NOX5*, which plays a role in cell growth and apoptosis (UniProt Consortium 2015). Another tumor suppressor gene, *TPM3*, was duplicated in the humpback whale assembly based on our gene annotation. However, these extranumerary copies of *TPM3* were not annotated on any humpback whale LSDs, lacked introns, and contained mostly the same exons, suggesting retrotransposition rather than segmental duplication as a mechanism for their copy number expansion (Kaessmann 2010). According to the RNA-Seq data, all seven copies of *TPM3* are expressed in humpback whale skin.

Duplications of the tumor suppressor gene *TP53* have been inferred as evidence for cancer suppression in elephants (Abegglen et al. 2015; Caulin et al. 2015; Sulak et al. 2016). During our initial scans for segmental duplications, we noticed a large pileup of reads in the MAKER-annotated humpback whale *TP53* (data not shown). We PCR-amplified, cloned, and sequenced this region from a humpback whale DNA sample, inferring four haplotypes that differ at two bases (supplementary Methods, Supplementary Material online). After manually annotating *TP53* in the humpback whale, we determined that these nucleotide variants fell in noncoding regions of the gene; one occurred upstream of the start codon whereas the other occurred between the first and second coding exons. Other genomic studies have concluded that *TP53* is not duplicated in cetaceans (Yim et al. 2014; Keane et al. 2015; Sulak et al. 2016). We consider the possibility of at least two *TP53* homologs in the genome of the humpback whale, although more data are required to resolve this. Regardless, cancer suppression likely arose in different mammalian lineages via multiple molecular etiologies. Overall, our results reveal several copy number expansions in cetaceans related to immunity, aging, and cancer, suggesting that cetaceans are among the large mammals that have evolved specific adaptations related to cancer resistance.

### Accelerated Regions in Cetacean Genomes Are Significantly Enriched with Pathways Relevant to Cancer

In order to determine genomic loci underlying cetacean adaptations, we estimated regions in the 12-mammal WGA with elevated substitution rates that were specific to the cetacean branches of the mammalian phylogeny. These genomic regions departed from neutral expectations in a manner consistent with either positive selection or relaxed purifying selection along the cetacean lineage (Pollard et al. 2010). We successfully mapped 3,260 protein-coding genes with functional annotations that overlap cetacean-specific accelerated regions, which were significantly enriched for Gene Ontology (GO) categories such as cell-cell signaling (GO:0007267) and cell adhesion (GO:0007155) (table 4). Adaptive change in cell signaling pathways could have maintained the ability of cetaceans to prevent neoplastic progression as they evolved larger body sizes. Adhesion molecules are integral to the development of cancer invasion and metastasis, and these results suggest that cetacean evolution was accompanied by selection pressure changes on both intra- and extracellular interactions. Cetacean-specific genomic regions with elevated substitution rates were also significantly enriched in genes involved in B-cell-mediated immunity (GO:0019724), likely due to the important role of regulatory cells which modulate immune response to not only pathogens but perhaps tumors as well. In addition, cetacean-specific acceleration in regions controlling complement activation (GO:0006956) may have provided better immunosurveillance against cancer and further protective measures against malignancies (Pio et al. 2014). We also found that accelerated regions in cetacean genomes were significantly enriched for genes controlling sensory perception of smell (GO:0007608), perhaps due to the relaxation of purifying selection in olfactory regions, which were found to be underrepresented in cetacean genomes (Yim et al. 2014).

**Table 4.** GO Terms for Biological Processes Overrepresented by Genes Overlapping Genomic Regions with Elevated Substitution Rates That Are Unique to the Cetacean Lineage.

Go Term	Description	Number of Genes	Fold Enrichment	***P*-Value** ^a^
GO:0007608	Sensory perception of smell	157	4.58	1.24E-40
GO:0006956	Complement activation	39	2.91	4.91E-05
GO:0019724	B-cell-mediated immunity	39	2.91	4.91E-05
GO:0032989	G-protein-coupled receptor signaling pathway	159	2.44	1.96E-17
GO:0042742	Defense response to bacterium	39	2.44	2.20E-03
GO:0009607	Response to biotic stimulus	43	2.09	1.85E-02
GO0007155	Cell adhesion	101	1.99	1.74E-06
GO:0007267	Cell–cell signaling	137	1.69	2.84E-05

a After Bonferonni correction for multiple testing.

### Selection Pressures on Protein-Coding Genes during Cetacean Evolution Point to Many Cetacean Adaptations, Including Cancer Suppression

**Fig. 3.**
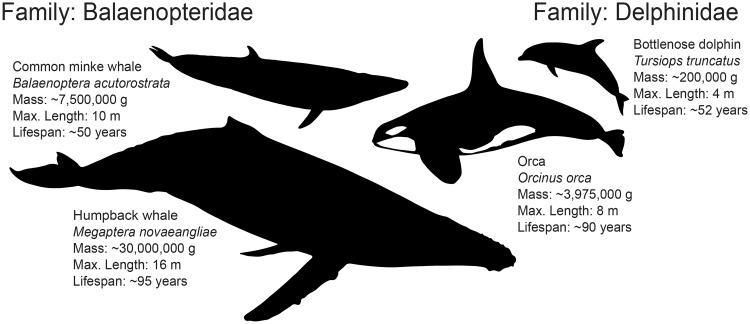
Diversity in both body size and lifespan within rorqual baleen whales (Balaenopteridae) and dolphins (Delphinidae). Maximal pairings using phylogenetic targeting (Arnold and Nunn 2010) of genome assembly-enabled cetaceans resulted in the most extreme divergence in both body size and lifespan between humpback whale (*Megaptera novaeangliae*) and common minke whale (*Balaenoptera acutorostrata*) within the Balaenopteridae, facing right, and orca (*Orcinus orca*) and bottlenose dolphin (*Tursiops truncatus*) within the Delphinidae, facing left. Trait data were collected from the panTHERIA (Jones et al. 2009) and AnAge (Tacutu et al. 2012) databases.

To gain further insight into the genomic changes underlying the evolution of large body sizes in cetaceans, we employed phylogenetic targeting to maximize statistical power in pairwise evolutionary genomic analyses (Arnold and Nunn 2010). This resulted in maximal comparisons between 1) the orca and the bottlenose dolphin and 2) the humpback whale and common minke whale. Despite their relatively recent divergences (e.g., the orca:bottlenose dolphin divergence is similar in age to that of the human:chimpanzee divergence, see fig. 2), the species pairs of common minke:humpback and orca:dolphin have each undergone extremely divergent evolution in body size and longevity (fig. 3). Humpback whales are estimated to weigh up to four times as much as common minke whales and are reported to have almost double the longevity, and orcas may weigh almost 20 times as much as bottlenose dolphins, also with almost double the lifespan (Tacutu et al. 2012). In order to offset the tradeoffs associated with the evolution of large body size, with the addition of many more cells and longer lifespans since the divergence of each species pair, we hypothesize that necessary adaptations for cancer suppression should be encoded in the genomes, as predicted by Peto’s Paradox (Tollis et al. 2017).

For each pairwise comparison, we inferred pairwise genome alignments with the common minke whale and orca genome assemblies as targets, respectively, and extracted protein-coding orthologous genes. We then estimated the ratio of nonsynonymous substitutions per synonymous site to synonymous substitutions per synonymous site (*d*_N_/*d*_S_) in order to measure selective pressures acting on each orthologous gene pair during cetacean evolution. A *d*_N_/*d*_S_ >1 is used to infer potentially functional amino acid changes in candidate genes subjected to positive selection (Fay and Wu 2003). Among an estimated 435 genes with *d_N_/d_S_* >1 in the common minke:humpback pairwise comparison, we detected eight genes belonging to the *JAK-STAT* signaling pathway (3.9-fold enrichment, *P* = 1.1E-3 Fisher’s exact test) and seven involved in cytokine–cytokine receptor interaction (4.1-fold enrichment, *P* = 1.7E-2 Fisher’s exact test) suggesting positive selection acting on pathways involved in cell proliferation. These genes included multiple members of the tumor necrosis factor subfamily such as *TNFSF15*, which inhibits angiogenesis and promotes the activation of caspases and apoptosis (Yu et al. 2001). A *d*_N_/*d*_S_ >1 was also detected in seven genes involved in the negative regulation of cell growth (GO:0030308, 3.1-fold enrichment, *P* = 8.03E-3 Fisher’s exact test), and five genes involved in double-strand break repair (GO:0006302, 4.0-fold enrichment, *P* = 8.03E-3 Fisher’s exact test). Although these results suggest the evolution of amino acid differences since the split between common minke and humpback whales in genes affecting cell growth, proliferation, and maintenance, the GO category enrichment tests did not pass significance criteria after Bonferroni corrections for multiple testing. We found 18 genes that are mutated in cancers according to the COSMIC v85 database (Forbes et al. 2015) in the common minke:humpback comparison, including a subset of five annotated as tumor suppressor genes, oncogenes, or fusion genes in the Cancer Gene Census (CGC; Futreal et al. 2004) which are highlighted in table 5. The complete list of COSMIC genes with elevated *d*_N_/*d*_S_ in the pairwise comparisons is given in supplementary table 8, Supplementary Material online. We detected 555 orthologous genes with *d*_N_/*d*_S_ >1 in the orca:dolphin comparison, which are significantly enriched (after Bonferroni correction for multiple testing) for biological processes such as immune response, cell activation, and regulation of cytokines (table 6), and 41 of which are known cancer genes according to COSMIC and CGC (table 5, supplementary table 8, Supplementary Material online). These results are consistent with our accelerated region analysis based on the WGA, which showed accelerated evolution in immunity pathways (above, see table 4). For instance, eight genes (*CD58*, *CD84*, *KLF13*, *SAMSN1*, *CTSG*, *GPC3*, *LTF*, and *SPG21*) annotated for immune system process (GO:0002376) were found in cetacean-specific accelerated genomic regions and also had a pairwise *d*_N_/*d*_S_ >1 in the orca:dolphin comparison, mirroring other recent genomic analyses of immunity genes in orcas (Ferris et al. 2018). Our results also suggest that the evolution of gigantism and long lifespans in cetaceans was accompanied by selection acting on many genes related to somatic maintenance and cell signaling.

**Table 5.** CGC Genes with *d*_N_/*d*_S_ > 1 as Revealed by Pairwise Comparisons of Cetacean Genomes.

Comparison	Gene Symbol	Gene Name	Role in Cancer	Function
Minke:humpback	CD274	CD274 molecule	TSG	Plays a critical role in induction and maintenance of immune tolerance to self^a^
	ETNK1	Ethanolamine kinase 1	TSG	Suppresses escaping of programmed cell death^b^
	IL21R	Interleukin 21 receptor	Fusion	The ligand binding of this receptor leads to the activation of multiple downstream signaling molecules, including JAK1, JAK3, STAT1, and STAT3.2
	MYOD1	Myogenic differentiation 1	Fusion	Regulates muscle cell differentiation by inducing cell cycle arrest, a prerequisite for myogenic initiation^a^
	PHF6	PHD finger protein 6	TSG	Encodes a protein with two PHD-type zinc finger domains, indicating a potential role in transcriptional regulation, that localizes to the nucleolus^a^
Orca:dolphin	BTG1	B-cell translocation gene 1; antiproliferative	TSG; fusion	Member of an antiproliferative gene family that regulates cell growth and differentiation^a^
	CD274	CD274 molecule	TSG; fusion	Plays a critical role in induction and maintenance of immune tolerance to self^a^
	FANCD2	Fanconi anemia; complementation group D2	TSG	Suppresses genome instability and mutations; promotes escaping programmed cell death; suppresses proliferative signaling; suppresses invasion and metastasis^b^
	FAS	Fas cell surface death receptor	TSG	Promotes cell replicative immortality; promotes proliferative signaling; promotes invasion and metastasis; suppresses escaping programmed cell death^b^
	FGFR4	Fibroblast growth factor receptor 4	Oncogene	Promotes proliferative signaling; promotes invasion and metastasis^b^
	GPC3	Glypican 3	Oncogene; TSG	Promotes invasion and metastasis; promotes suppression of growth^b^
	HOXD11	Homeobox D11	Oncogene; fusion	The homeobox genes encode a highly conserved family of transcription factors that play an important role in morphogenesis in all multicellular organisms^a^
	HOXD13	Homeobox D13	Oncogene; fusion	
	LASP1	LIM and SH3 protein 1	Fusion	The encoded protein has been linked to metastatic breast cancer, hematopoetic tumors such as B-cell lymphomas, and colorectal cancer^a^
	MLF1	Myeloid leukemia factor 1	TSG; fusion	This gene encodes an oncoprotein which is thought to play a role in the phenotypic determination of hemopoetic cells. Translocations between this gene and nucleophosmin have been associated with myelodysplastic syndrome and acute myeloid leukemia^a^
	MYB	v-myb myeloblastosis viral oncogene homolog	Oncogene; fusion	This gene may be aberrantly expressed or rearranged or undergo translocation in leukemias and lymphomas, and is considered to be an oncogene^a^
	MYD88	Myeloid differentiation primary response gene (88)	Oncogene	Promotes escaping programmed cell death; promotes proliferative signaling; promotes invasion and metastasis; promotes tumor promoting inflammation^b^
	NR4A3	Nuclear receptor subfamily 4; group A; member 3 (NOR1)	Oncogene; fusion	Encodes a member of the steroid–thyroid hormone–retinoid receptor superfamily that may act as a transcriptional activator^a^
	PALB2	Partner and localizer of BRCA2	TSG	This protein binds to and colocalizes with the breast cancer 2 early onset protein (BRCA2) in nuclear foci and likely permits the stable intranuclear localization and accumulation of BRCA2^a^
	PML	Promyelocytic leukemia	TSG; fusion	Expression is cell-cycle related and it regulates the p53 response to oncogenic signals^a^
	RAD21	RAD21 homolog (*Schizosaccharomyces pombe*)	Oncogene; TSG	Promotes invasion and metastasis; suppresses genome instability and mutations; suppresses escaping programmed cell death^b^
	STIL	SCL/TAL1 interrupting locus	Oncogene; fusion	Encodes a cytoplasmic protein implicated in regulation of the mitotic spindle checkpoint, a regulatory pathway that monitors chromosome segregation during cell division to ensure the proper distribution of chromosomes to daughter cells^a^
	TAL1	T-cell acute lymphocytic leukemia 1 (SCL)	Oncogene; fusion	Implicated in the genesis of hemopoietic malignancies and may play an important role in hemopoietic differentiation^a^
	TNFRSF14	Tumor necrosis factor receptor superfamily; member 14 (herpesvirus entry mediator)	TSG	The encoded protein functions in signal transduction pathways that activate inflammatory and inhibitory T-cell immune response^a^
	TNFRSF17	Tumor necrosis factor receptor superfamily; member 17	Oncogene; fusion	This receptor also binds to various TRAF family members, and thus may transduce signals for cell survival and proliferation^a^

Note.—TSG, tumor suppressor gene.

a Source: RefSeq.

b Source: Cancer Hallmark from CGC.

**Table 6.** GO Terms for Biological Processes Overrepresented by Genes with Pairwise *d*_N_/*d*_S_ > 1 in the Orca: Bottlenose Dolphin Comparison.

GO Term	Description	Number of Genes	Fold Enrichment	***P*-Value** ^a^
GO:0031347	Regulation of defense response	36	2.70	2.91E-2
GO:0050776	Regulation of immune response	50	2.18	1.95E-3
GO:0002694	Regulation of leukocyte activation	31	2.49	1.89E-2
GO:0002275	Myeloid cell activation involved in immune response	29	2.53	2.37E-2
GO:0002699	Positive regulation of immune effector process	19	4.46	1.01-E3
GO:0042108	Positive regulation of cytokine biosynthetic process	9	6.87	2.23E-2

a After Bonferonni correction for multiple testing.

**Fig. 4.**
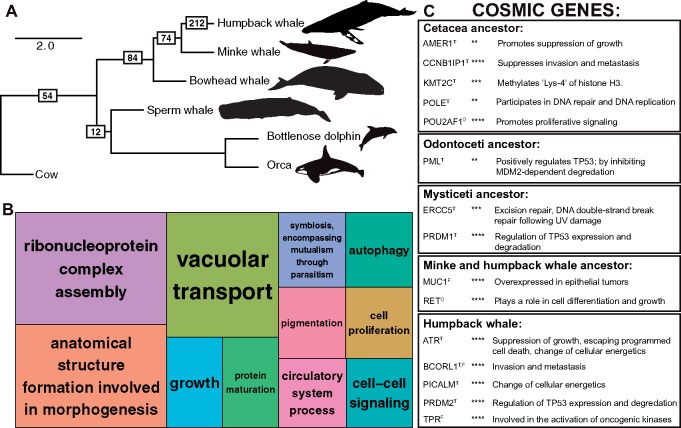
Positively selected genes during cetacean evolution. (*A*) Species tree relationships of six modern cetaceans with complete genome assemblies, estimated from 152 single-copy orthologs. Branch lengths are given in coalescent units. Outgroup taxa are not shown. The complete species tree of 28 mammals is shown in supplementary figure 4, Supplementary Material online. Boxes with numbers indicate the number of positively selected genes passing filters and a Bonferroni correction detected on each branch. (*B*) TreeMap from REVIGO for GO biological processes terms represented by genes evolving under positive selection across all cetaceans. Rectangle size reflects semantic uniqueness of GO term, which measures the degree to which the term is an outlier when compared semantically to the whole list of GO terms. (*C*) Cancer gene names and functions from COSMIC found to be evolving under positive selection in the cetacean branch-site models. Superscripts for gene names indicate as follows: T, tumor suppressor gene; O, oncogene; F, fusion gene. Asterisks indicate *P*-value following FDR correction for multiple testing: **P < 0.01, ***P < 0.001, ****P < 0.0001.

As a more accurate assessment of selection pressure variation acting on protein-coding genes across cetacean evolution, we conducted an additional assessment of *d*_N_/*d*_S_ using branch-site codon models implemented in codeml (Yang 1998). We employed extensive filtering of the branch site results, including both false discovery rate (FDR) and Bonferroni corrections for multiple testing (see Materials and methods), and conservatively estimated that 450 protein-coding genes were subjected to positive selection in cetaceans. These include 54 genes along the ancestral cetacean branch, 12 along the ancestral toothed whale branch, 84 along the ancestral baleen whale branch, 74 in the ancestor of common minke and humpback whales, and 212 unique to the humpback whale branch (fig. 4*A*). Cetacean positively selected genes were annotated for functions related to extensive changes in anatomy, growth, cell signaling, and cell proliferation (fig. 4*B*). For instance, in the branch-site models for humpback whale, positively selected genes are enriched for several higher-level mouse limb phenotypes including those affecting the limb long bones (MP:0011504, 15 genes, FDR-corrected *P*-value = 0.001), and more specifically the hind limb stylopod (MP:0003856, seven genes, FDR = 0.024) or femur (MP:0000559, six genes, FDR = 0.019). These phenotypes are reminiscent of the developmental basis of hind limb loss in cetaceans; embryonic studies show that hind limb buds are initially formed but disappear by the fifth gestational week (Thewissen et al. 2006). Enriched mouse phenotypes are also related to the unique cetacean axial skeleton (MP:0002114, 25 genes, FDR = 0.016), most notably in the skull, including craniofacial bones (MP:0002116, 17 genes, FDR = 0.018), teeth (MP:0002100, nine genes, FDR = 0.004), and the presphenoid (MP:0030383, three genes, FDR = 0.003). Past analyses of the cetacean basicranial elements revealed that the presphenoid was extensively modified along the cetacean lineage (Ichishima 2016).

Positively selected genes unique to the humpback whale were significantly enriched for a single biological process: regulation of cell cycle checkpoint (GO:1901976; 18.57-fold enrichment, *P* = 0.02 after Bonferonni correction for multiple testing), suggesting positive selection in pathways that control responses to endogenous or exogenous sources of DNA damage and limit cancer progression (Kastan and Bartek 2004). We detected a significant number of protein–protein interactions among humpback whale-specific positively selected genes (number of nodes = 204, number of edges = 71, expected number of edges = 51, *P* = 0.004; supplementary fig. 10, Supplementary Material online), including genes that are often coexpressed and involved in DNA repair, DNA replication, and cell differentiation. For instance, we identified significant interactions between *DNA2*, which encodes a helicase involved in the maintenance of DNA stability, and *WDHD1* which acts as a replication initiation factor. Another robust protein interaction network was detected between a number of genes involved in the genesis and maintenance of primary cilia. The highest scoring functional annotation clusters resulted in key words such as ciliopathy (seven genes) and cell projection (16 genes), and GO terms such as cilium morphogenesis, cilium assembly, ciliary basal body, and centriole. The primary cilia of multicellular eukaryotes control cell proliferation by mediating cell-extrinsic signals and regulating cell cycle entry, and defects in ciliary regulation are common in many cancers (Michaud and Yoder 2006).

Our branch-site test results indicated that the evolution of cetacean gigantism was accompanied by strong selection on many pathways that are directly linked to cancer (fig. 4*C*). We identified 33 genes that are mutated in human cancers (according to the COSMIC database) that were inferred as subjected to positive selection in the humpback whale lineage, including the known tumor suppressor genes *ATR*, which is a protein kinase that senses DNA damage upon genotixic stress and activates cell cycle arrest, and *RECK*, which suppresses metastasis (Forbes et al. 2015). Multiple members of the PR domain-containing gene family (PRDM) evolved under positive selection across cetaceans, including the tumor suppressor genes *PRDM1*, whose truncation leads to B-cell malignancies, and *PRDM2*, which regulates the expression and degradation of *TP53* (Shadat et al. 2010) and whose forced expression causes apoptosis and cell cycle arrest in cancer cell lines (Fog et al. 2012). In baleen whales, *ERCC5*, which is a DNA repair protein that partners with *BRCA1* and *BRCA2* to maintain genomic stability (Trego et al. 2016) and suppresses UV-induced apoptosis (Clément et al. 2006), appeared to have been subjected to positive selection as well. Across all the branch-site models, positively selected genes represented multiple functional categories relevant to cancer and Peto’s Paradox.

Among the cancer-related genes subjected to positive selection in cetaceans, we identified two with identical amino acid changes among disparate taxa united by the traits of large body size and/or extreme longevity. Specifically, *PRDM13* is a tumor suppressor gene that acts as a transcriptional repressor, and we found identical D→E amino acid substitutions in this gene in sperm whale, dolphin, orca, and humpback but also manatee (*Trichechus manatus*) and African elephant (*Loxodonta africana*) which are large-bodied afrotherian mammals that have been the focus of cancer suppression research (Abegglen et al. 2015; Sulak et al. 2016). Secondly, *POLE* is a cancer-related gene that participates in DNA repair and replication, and we observed one I→V substitution shared among orca, dolphin, bowhead, humpback, and common minke whale, but also elephant, as well as a second I→V substitution shared with these cetaceans and the little brown bat (*Myotis lucifugus*). Vesper bats such as *M. lucifugus* are known for their exceptional longevity relative to their body size, and have been proposed as model organisms in senescence and cancer research (Foley et al. 2018). Parallel changes in cancer-related genes across these phylogenetically distinct mammals suggest natural selection has acted on similar pathways that limit neoplastic progression in large and long-lived species (Tollis et al. 2017).

### Peto’s Paradox and Cancer in Whales and Other Large Mammals

Large body size has evolved numerous times in mammals, and although it is exemplified in some extant cetaceans, gigantism is also found in afrotherians, perissodactyls, and carnivores (Baker et al. 2015). Our results suggest that cancer suppression in large and long-lived mammals has also evolved numerous times. However, none of these species is completely immune to cancer. Elephants have at least a 5% lifetime risk of cancer mortality (Abegglen et al. 2015), which is far less than humans, but detecting cancer, and estimating cancer incidence and mortality rates in wild cetaceans is more challenging. Mathematical modeling predicting the lifetime risk of colorectal cancer in mice and humans yielded a rate of colorectal cancer at 50% in blue whales by age 50, and 100% by age 90 (Caulin et al. 2015). This high rate of cancer mortality is an unlikely scenario, and taken with our genomic results presented here it suggests that cetaceans have evolved mechanisms to limit their overall risk of cancer. Among baleen whales, benign neoplasms of the skin, tongue, and central nervous system have been reported in humpback whales, and ovarian carcinomas and lymphomas have been detected in fin whales (Newman and Smith 2006). Among smaller cetaceans, one unusually well-documented case study concluded that 27% of beluga whales (*Delphinapterus leucas*) found dead in the St. Lawrence estuary had cancer, which may have contributed to 18% of the total mortality in that population (Martineau et al. 2002). The authors suggested that the high degree of polycyclic aromatic hydrocarbons released into the estuary by nearby industry may have contributed to this elevated cancer risk (Martineau et al. 2002). By contrast, the larger baleen whale species in the Gulf of St. Lawrence appear to have lower contaminant burdens, likely due to ecological differences (Gauthier et al 1997). Interestingly, unlike in human cells, homologous recombination is uninhibited in North Atlantic right whale lung cells following prolonged exposure to the human lung carcinogen particulate hexavalent chromium (Browning et al. 2017), suggesting adaptations for high-fidelity DNA repair in whales.

In this study, we provide a de novo reference assembly for the humpback whale—one of the more well-studied giants living on Earth today. The humpback whale genome assembly is highly contiguous and contains a comparable number of orthologous genes to other mammalian genome projects. Our comparisons with other complete cetacean genomes confirm the results of other studies which concluded that rorqual whales likely began diversifying during the Miocene (Slater et al. 2017; Árnason et al. 2018). We found indications of positive selection on many protein-coding genes suggestive of adaptive change in pathways controlling the mammalian appendicular and cranial skeletal elements, which are relevant to highly specialized cetacean phenotypes, as well as in many immunity genes and pathways that are known to place checks on neoplastic progression. LSDs in cetacean genomes contain many genes involved in the control of apoptosis, including known tumor suppressor genes, and skin transcriptome results from humpback whale suggest many gene duplications, whether through segmental duplication or retrotransposition, are transcribed and hence likely functional. We also use genome-wide evidence to show that germline mutation rates may be slower in cetaceans than in other mammals, which has been suggested in previous studies (Jackson et al. 2009), and we suggest as a corollary that cetacean somatic mutations rates may be lower as well. These results are consistent with predictions stemming from Peto’s Paradox (Peto et al. 1975; Caulin and Maley 2011), which posited that gigantic animals have evolved compensatory adaptations to cope with the negative effects of orders of magnitude more cells and long lifespans that increase the number of cell divisions and cancer risk over time. Altogether, the humpback whale genome assembly will aid comparative oncology research that seeks to improve therapeutic targets for human cancers, as well as provide a resource for developing useful genomic markers that will aid in the population management and conservation of whales.

## Materials and Methods

### Tissue Collection and DNA Extraction

Biopsy tissue was collected from an adult female humpback whale (“Salt,” NCBI BioSample SAMN1058501) in the Gulf of Maine, western North Atlantic Ocean using previously described techniques (Lambertsen 1987; Palsbøll et al. 1991) and flash frozen in liquid nitrogen. We extracted DNA from skin using the protocol for high-molecular-weight genomic DNA isolation with the DNeasy Blood and Tissue purification kit (Qiagen). Humpback whales can be individually identified and studied over time based on their unique ventral fluke pigmentation (Katona and Whitehead 1981). Salt was specifically selected for this study because of her 35-year prior sighting history, which is among the lengthiest and detailed for an individual humpback whale (Center for Coastal Studies, unpublished data).

### De Novo Assembly of the Humpback Whale Genome

Using a combination of paired-end and mate-pair libraries, de novo assembly was performed using Meraculous 2.0.4 (Chapman et al. 2011) with a kmer size of 47. Reads were trimmed for quality, sequencing adapters, and mate-pair adapters using Trimmomatic (Bolger et al. 2014). The genome size of the humpback whale was estimated using the short reads, by counting the frequency of kmers of length 27 occurring in the 180-bp data set, estimating the kmer coverage, and using the following formula: genome size = total kmers ÷ kmer coverage.

### Chicago Library Preparation and Sequencing

Four Chicago libraries were prepared as described previously (Putnam et al. 2016). Briefly, for each library, ∼500 ng of high-molecular-weight genomic DNA (mean fragment length >50 kb) was reconstituted into chromatin in vitro and fixed with formaldehyde. Fixed chromatin was digested with *Mbo*I or *Dpn*II, the 5′-overhangs were repaired with biotinylated nucleotides, and blunt ends were ligated. After ligation, crosslinks were reversed and the DNA purified from protein. Biotin that was not internal to ligated fragments was removed from the purified DNA. The DNA was then sheared to ∼350 bp mean fragment size and sequencing libraries were generated using NEBNext Ultra (New England BioLabs) enzymes and Illumina-compatible adapters. Biotin-containing fragments were isolated using streptavidin beads before PCR enrichment of each library. The libraries were sequenced on an Illumina HiSeq 2500 platform.

### Scaffolding the De Novo Assembly with HiRise

The input de novo assembly, shotgun reads, and Chicago library reads were used as input data for HiRise, a software pipeline designed specifically for using Chicago data to scaffold genome assemblies (Putnam et al. 2016). Shotgun and Chicago library sequences were aligned to the draft input assembly using a modified SNAP read mapper (http://snap.cs.berkeley.edu). The separations of Chicago read pairs mapped within draft scaffolds were analyzed by HiRise to produce a likelihood model for genomic distance between read pairs, and the model was used to identify putative misjoins and to score prospective joins. After scaffolding, shotgun sequences were used to close gaps between contigs.

### Assessing the Gene Content of the Humpback Whale Assembly

The expected gene content of the assembly was evaluated using the Core Eukaryotic Genes Mapping Approach (Parra et al. 2009) which searches the assembly for 458 highly conserved proteins and reports the proportion of 248 of the most highly conserved orthologs that are present in the assembly. We also used the Benchmarking Universal Single Copy Orthologs (BUSCO v2.0.1; Simão et al. 2015), which analyzes genome assemblies for the presence of 3,023 genes conserved across vertebrates, as well as a set of 6,253 genes conserved across laurasiatherian mammals.

### Transcriptome Sequencing and Assembly

In order to aid in our gene-finding efforts for the humpback whale genome assembly and to measure gene expression, we generated transcripts from skin tissue by extracting total RNA using the QIAzol Lysis Reagent (Qiagen), followed by purification on RNeasy spin columns (Qiagen). RNA integrity and quantity were determined on the Agilent 2100 Bioanalyzer (Agilent) using the manufacturer’s protocol. The total RNA was treated with DNase using DNase mix from the RecoverAll Total Nucleic Acid Isolation kit (Applied Biosystems/Ambion). The RNA library was prepared and sequenced by the Genome Technology Center at the University of California Santa Cruz, including cDNA synthesis with the Ovation RNA-Seq system V2 (Nugen) and RNA amplification as described previously (Tariq et al. 2011). We used 0.5–1 µg of double-stranded cDNA for library preparation, sheared using the Covaris S2 size-selected for 350–450 bp using automated electrophoretic DNA fractionation system (LabChipXT, Caliper Life Sciences). Paired-end sequencing libraries were constructed using Illumina TruSeq DNA Sample Preparation Kit. Following library construction, samples were quantified using the Bioanalyzer and sequenced on the Illumina HiSeq 2000 platform to produce 2 × 100 bp sequencing reads. We then used Trinity (Grabherr et al. 2011) to assemble the adapter-trimmed RNA-Seq reads into transcripts.

### Genome Annotation

We generated gene models for the humpback whale using multiple iterations of MAKER2 (Holt and Yandell 2011) which incorporated 1) direct evidence from the Trinity-assembled transcripts, 2) homology to NCBI proteins from ten mammals (human, mouse, dog, cow, sperm whale, bottlenose dolphin, orca, bowhead whale, common minke whale, and baiji) and UniProtKB/Swiss-Prot (UniProt Consortium 2015), and 3) ab initio gene predictions using SNAP (11/29/2013 release; Korf 2004) and Augustus v3.0.2 (Stanke et al. 2008). A detailed description of the annotation pipeline is provided in the supplementary Methods, Supplementary Material online. Final gene calls were annotated functionally by BlastP similarity to UniProt proteins (UniProt Consortium 2015) with an *e*-value cutoff of 1e-6.

### Repeat Annotation and Evolutionary Analysis

To analyze the repetitive landscape of the humpback whale genome, we used both database and de novo modeling methods. For the database method, we ran RepeatMasker v4.0.5 (http://www.repeatmasker.org, accessed August 21, 2017) (Smit et al. 2015a) on the final assembly, indicating the “mammalia” repeat library from RepBase (Jurka et al. 2005). For the de novo method, we scanned the assembly for repeats using RepeatModeler v1.0.8 (http://www.repeatmasker.org) (Smit et al. 2015b), the results of which were then classified using RepeatMasker. To estimate evolutionary divergence within repeat subfamilies in the humpback whale genome, we generated repeat-family-specific alignments and calculated the average Kimura-2-parameter divergence from consensus within each family, correcting for high mutation rates at CpG sites with the calcDivergenceFromAlign.pl RepeatMasker tool. We compared the divergence profile of humpback whale and bowhead whale by completing parallel analyses, and the repetitive landscapes of orca and bottlenose dolphin are available from the RepeatMasker server (http://www.repeatmasker.org/species, accessed August 21, 2017).

### Analysis of Gene Expression Using RNA-Seq

Splice-wise mapping of RNA-Seq reads against the humpback whale genome assembly and annotation was carried out using STAR v2.4 (Dobin et al. 2013), and we counted the number of reads mapping to gene annotations. We also mapped the skin RNA-Seq data to the database of annotated humpback whale transcripts using local alignments with bowtie v2.2.5 (Langmead and Salzberg 2012), and used stringtie v1.3.4 (Pertea et al. 2015) to calculate gene abundances by transcripts per million.

### Analysis of Segmental Duplications in Cetacean Genomes

In order to detect LSDs in several cetacean genomes, we applied an approach based on depth of coverage (Alkan et al. 2009). To this end, we used whole-genome shotgun sequence data from the current study as well as from other cetacean genomics projects. All data were mapped against the humpback whale reference assembly. A detailed description of the segmental duplication analysis is provided in the supplementary Methods, Supplementary Material online.

### Whole-Genome Alignments

We generated WGAs of 12 mammals (supplementary table 2, Supplementary Material online). First, we generated pairwise syntenic alignments of each species as a query to the human genome (hg19) as a target using LASTZ v1.02 (Harris 2007), followed by chaining to form gapless blocks and netting to rank the highest scoring chains (Kent et al. 2003). The pairwise alignments were used to construct a multiple sequence alignment with MULTIZ v11.2 (Blanchette et al. 2004) with human as the reference species. We filtered the MULTIZ alignment to only contain aligned blocks from at least 10 out of the 12 species (81% complete).

### Phylogenetic Reconstruction Using Single-Copy Orthologs

We downloaded the coding DNA sequences from 28 publicly available mammalian genome assemblies (supplementary table 9, Supplementary Material online) and used VESPA (Webb et al. 2017) to obtain high-confidence SGOs (supplementary Methods, Supplementary Material online). For phylogenetic analysis, we filtered the SGO data set to include only loci that were represented by at least 24 out of the 28 mammalian species (86% complete) and reconstructed each gene tree using maximum likelihood in PhyML v3.0 (Guindon et al. 2010) with an HKY85 substitution model and 100 bootstrap replicates to assess branch support. The gene trees were then binned and used to reconstruct a species tree using the accurate species tree algorithm (ASTRAL-III v5.6; Zhang et al. 2018). ASTRAL utilizes the multispecies coalescent model that incorporates incomplete lineage sorting, and finds the species tree stemming from bipartitions predefined by the gene trees. Branch support for the species tree was assessed with local posterior probabilities, and branch lengths were presented in coalescent units, where shorter branch lengths indicate greater gene tree discordance (Sayyari and Mirarab 2016).

### Rates of Molecular Evolution and Divergence Time Estimation

We used multiple approaches on independent data sets to estimate rates of molecular evolution and the divergence times of the major mammalian lineages including six modern whales with complete genome assemblies. We first focused on 4-fold degenerate (4D) sites, which are positions within codon alignments where substitutions result in no amino acid change and can be used to approximate the neutral rate of evolution (Kumar and Subramanian 2002). We used the Ensembl human gene annotation to extract coding regions from the 12-mammal WGA using msa_view in PHAST v1.4 (Hubisz et al. 2011). We reconstructed the phylogeny with the 4D data as a single partition in RAxML v8.3 (Stamatakis 2014) under the GTRGAMMA substitution model and assessed branch support with 10,000 bootstraps. Rates of molecular evolution were estimated on the 4D data set with the semiparametric PL method implemented in r8s v1.8 (Sanderson 2002, 2003). A detailed description of the PL method is given in the supplementary Methods, Supplementary Material online.

We also used the approximate likelihood calculation in MCMCtree (Yang and Rannala 2006) to estimate divergence times using independent data sets: 1) the above-mentioned 4D data set derived from the WGA, as well as 2) a set of the SGOs that included 24 out of 28 sampled taxa (86%) and was partitioned into three codon positions. We implemented the HKY85 substitution model, multiple fossil-based priors (supplementary table 10, Supplementary Material online; Mitchell 1989; Benton et al. 2015; Hedges et al. 2015), and independent rates (“clock = 3”) along branches. All other parameters were set as defaults. For each MCMCtree analysis, we ran the analysis three times with different starting seeds and modified the Markov chain Monte Carlo (MCMC) length and sampling frequency in order to achieve proper chain convergence, monitored with Tracer v1.7. We achieved proper MCMC convergence on the 4D data set after discarding the first 500,000 steps as burn-in and sampling every 2,000 steps until we collected 20,000 samples. We achieved proper MCMC convergence on the SGO data set after discarding the first 500,000 steps as burn-in and sampling every 10,000 steps until we collected 10,000 samples.

### Demographic Analysis

We used the PSMC (Li and Durbin 2011) to reconstruct the population history of North Atlantic humpback whales, including the individual sequenced in the current study (downsampled to ∼20× coverage) and a second individual sequenced at ∼17× coverage in Árnason et al. (2018). A detailed description of the PSMC analysis is provided in the supplementary Methods, Supplementary Material online.

### Nonneutral Substitution Rates in Cetacean Genomes

In order to identify genomic regions controlling cetacean-specific adaptations, we used *phyloP* (Pollard et al. 2010) to detect loci in the 12-mammal WGA that depart from neutral expectations (see supplementary Methods, Supplementary Material online). We then collected accelerated regions that overlapped human whole gene annotations (hg19) using *bedtools intersect* (Quinlan and Hall 2010) and tested for the enrichment of GO terms using the PANTHER analysis tool available at the Gene Ontology Consortium website (GO Ontology database, last accessed June 2017) (Gene Ontology Consortium 2015).

### Detection of Protein-Coding Genes Subjected to Positive Selection

In order to measure selective pressures acting on protein-coding genes during cetacean evolution, with an emphasis on the evolution of cancer suppression, we estimated the ratio of nonsynonymous to synonymous substitutions (*d*_N_/*d*_S_). To maximize statistical power in pairwise comparisons given the number of available cetacean genomes (six, last accessed September 2017), we implemented phylogenetic targeting (Arnold and Nunn 2010) assuming a phylogeny from a mammalian supertree (Fritz et al. 2009). To select genome assemblies most suitable for assessing Peto’s Paradox, we weighted scores for contrasts with a lot of change in the same direction for both body mass and maximum longevity. Trait values were taken from panTHERIA (Jones et al. 2009), and we selected maximal pairings based on the standardized summed scores. We then generated pairwise genome alignments as described above based on the phylogenetic targeting results. For each pairwise genome alignment, we stitched gene blocks in Galaxy (Blankenberg et al. 2011) according to the target genome annotations, producing alignments of one-to-one orthologs, which were filtered to delete frameshift mutations and replace internal stop codons with gaps. We then estimated pairwise *d*_N_/*d*_S_ for every orthologous gene pair with KaKs_Calculator v2.0 (Wang et al. 2010). To link genes with *d*_N_/*d*_S_ >1 to potential phenotypes, we used orthologous human Ensembl gene IDs to collect GO terms in BioMart (Kinsella et al. 2011) and tested for enrichment of overrepresented GO terms.

We also used codon-based models to test for selective pressure variation along branches of the cetacean phylogeny in comparison to other mammal lineages, also known as the branch-site test (Yang 2007). First, the known species phylogeny (Morgan et al. 2013; Tarver et al. 2016) was pruned to correspond to the species present in each SGO family. SGO nucleotide alignments that contained more than seven species were analyzed for selective pressure variation: This is to reduce the risk of detecting false positives (Anisimova et al. 2001, 2002). In general, the branch-site test is a powerful yet conservative approach (Gharib and Robinson-Rechavi 2013), although model misspecification and alignment errors can greatly increase the number of false positives (Anisimova et al. 2001, 2002). Recent studies have concluded that many published inferences of adaptive evolution using the branch site test may be affected by artifacts (Venkat et al. 2018). Therefore, extensive filtering is necessary in order to make reasonably sound conclusions from results of the branch site test. A detailed description of all tested models and the filtering process are given in the supplementary Methods, Supplementary Material online. In total, 1,152 gene families were analyzed. We carried out the branch-site test using PAML v4.4e (Yang 2007). The following five branches were assessed as foreground: humpback whale, the most recent common ancestor (MRCA) of the common minke and humpback whales, MRCA of baleen whales, MRCA of toothed whales, and the MRCA of all whales (cetacean stem lineage). For each model, we kept all genes that met a significance threshold of *P* < 0.05 after a Bonferonni correction for multiple hypothesis testing using the total number of branch genes (five foreground branches*1,152 genes). We also corrected the raw *P*-values from the likelihood ratio tests of every gene by the FDR correction where *q* = 0.05 (Benjamini and Hochberg 1995). The Bonferroni correction is more conservative than the FDR but sufficient for multiple hypothesis testing of lineage-specific positive selection; in these cases, the FDR results in higher probabilities of rejecting true null hypotheses (Anisimova and Yang 2007). Therefore, we used Bonferroni-corrected results in downstream analyses but also report FDR-corrected *P*-values in figure 4*C*. Genes were identified based on the human ortholog (Ensembl gene ID), and we performed gene annotation enrichment analysis and functional annotation clustering with DAVID v6.8 (Huang et al. 2009a, 2009b), as well as semantic clustering of GO terms using REVIGO (Supek et al. 2011). We also searched for interactions of positively selected proteins using STRING v10.5 (Szklarczyk et al. 2017) with default parameters, and tested for the enrichment of overrepresented GO terms as above and for associated mouse phenotypes using *mod*PhEA (Weng and Liao 2017).

## Data Availability

All data that contributed to the results of the study are made publicly available. The genomic sequencing, RNA sequencing, as well as the genome assembly for humpback whale (GCA_004329385.1) are available under NCBI BioProject PRJNA509641. The gene annotation, orthologous gene sets, positive selection results, segmental duplication annotations, and whole-genome alignments used in this study are available at the Harvard Dataverse (https://doi.org/10.7910/DVN/ADHX1O).

## Supplementary Material

Supplementary data are available at *Molecular Biology and Evolution* online.

## Supplementary Material

msz099_Supplementary_Data
