# Supplementary material for: Return to the Sea, Get Huge, Beat Cancer: An Analysis of Cetacean Genomes Including an Assembly for the Humpback Whale (Megaptera novaeangliae)
Source: Mol Biol Evol. 2019 May 9;36(8):1746–63. doi: 10.1093/molbev/msz099 (PMC6657726; doi:10.1093/molbev/msz099)
Supplement: msz099_Supplementary_Data [file msz099_supplementary_data.pdf]

Return to the sea, get huge, beat cancer: an analysis of cetacean genomes including an assembly for the humpback whale (*Megaptera novaeangliae*)

Marc Tollis[1,2,3*], Jooke Robbins[4], Andrew E. Webb[5], Lukas F.K. Kuderna[6], Aleah F. Caulin[7], Jacinda D. Garcia[2], Martine Bèrubè[8], Nader Pourmand[9], Tomas Marques-Bonet[6,10,11,12], Mary J. O'Connell[13], Per J. Palsbøll[8†], Carlo C. Maley[1,2†]

[1]Biodesign Institute, Arizona State University, Tempe, AZ; [2]School of Life Sciences, Arizona State University, Tempe, AZ; [3]School of Informatics, Computing, and Cyber Systems, Northern Arizona University, Flagstaff, AZ; [4]Center for Coastal Studies, Provincetown, MA; [5]Temple University, Philadelphia, PA; [6]Institute of Evolutionary Biology (UPF-CSIC), PRBB, Dr. Aiguader 88, 08003 Barcelona, Spain; [7]University of Pennsylvania, Philadelphia, PA; [8]University of Groningen, Groningen, Netherlands; [9]University of California Santa Cruz, Santa Cruz, CA; [10]CNAG-CRG, Centre for Genomic Regulation (CRG), The Barcelona Institute of Science and Technology, Baldiri Reixac 4, Barcelona 08028, Spain; [11]Institució Catalana de Recerca i Estudis Avançats (ICREA), Passeig Lluís Companys 23, Barcelona, Catalonia 08010, Spain; [12]Institut Català de Paleontologia Miquel Crusafont, Universitat Autònoma de Barcelona, Edifici ICTA-ICP, c/ Columnes s/n, 08193 Cerdanyola del Vallès, Barcelona, Spain; [13]Computational and Molecular Evolutionary Biology Research Group, School of Life Sciences, University of Nottingham, NG7 2RD, United Kingdom

SUPPLEMENTARY MATERIALS

Supplementary Methods

Genome annotation

We generated gene models for the humpback whale using multiple iterations of MAKER2 (Holt and Yandell 2011) which incorporated (1) direct evidence from the Trinity-assembled transcripts, (2) homology to NCBI proteins from 10 mammals (human, mouse, dog, cow, sperm whale, bottlenose dolphin, orca, bowhead whale, common minke whale, and baiji) and UniProtKB/Swiss-Prot (UniProt Consortium 2015) and (3) *ab-initio* gene predictions using SNAP (11/29/2013 release; Korf 2004) and Augustus v3.0.2 (Stanke et al. 2008). The first MAKER iteration aligned the transcript and protein sequences to the assembly and predicted genes using SNAP to produce draft gene models. As *ab-initio* gene prediction benefits from the training of Hidden Markov Models (HMM) to each genome, we trained SNAP using a three-pass approach in MAKER. First, we used the HMM generated from the CEGMA analysis to create initial models for SNAP as suggested in the MAKER documentation (last accessed June 2016), and ran SNAP with the evidence alignments in the first MAKER iteration. Second, the gene models generated from the first iteration were used to improve the SNAP HMM, which was then used in a second MAKER2 iteration. This process was repeated again to further to improve the predicted gene models. In parallel, Augustus HMMs from the BUSCO analysis were obtained. Finally, we ran the fully trained SNAP gene models, the aligned transcript and protein data, and the Augustus HMM to obtain the final gene models. To select the highest quality genome annotation possible given the data, we compared the annotation editing distance (Eilbeck et al. 2009) of annotation versions whose *ab initio* gene predictions utilized only the SNAP gene models, only the Augustus gene models, and both SNAP and Augustus gene models. Final gene calls were annotated functionally by BLASTp similarity to UniProt proteins (UniProt Consortium 2015) with and e-value cutoff of 1e-6.

To assess the quality of annotations that used varying degrees of the three types of evidence (expression, protein homology, and *ab initio* gene prediction), we used annotation editing distances (AED), which measure the difference between a gene call and its aligned evidence (an AED closer to zero requires less manual annotation)

(Yandell and Ence 2012). While the cumulative distribution frequencies of annotation editing distances (AED) showed >90% of the gene calls with AED <0.5 in all annotation versions, the annotation utilizing both SNAP (Korf 2004) and Augustus (Stanke et al. 2008) *ab initio* gene models resulted in more gene calls with lower AED (Supplementary Figure 1); therefore we used this version in all downstream analyses.

Analysis of segmental duplications in cetacean genomes

In order to detect large segmental duplications in several cetacean genomes, we applied an approach based on depth of coverage (Alkan et al. 2009). To this end, we used whole genome shotgun sequence data from the current study as well as from other cetacean genomics projects including the blue whale (*Balaenoptera musculus*), sei whale (*Balaenoptera borealis*), grey whale (*Eschrichtius robustus*), fin whale (*Balaenoptera physalus*), humpback whale, common minke whale (*Balaenoptera acutorostrata*), North Atlantic right whale (*Eubalaena glacialis*) (Árnason et al. 2018) (BioProject PRJNA389516), sperm whale (*Physeter macrocephalus*) (Warren et al. 2017), bowhead whale (*Balaena mysticetus*) (Keane et al. 2015), bottlenose dolphin (*Tursiops truncatus*) and orca (*Orcinus orca*) (Foote et al. 2015). All data was mapped against the humpback whale reference assembly. In order to detect copy number, the assembly was prepared as follows: We hard-masked all common repeats annotated by RepeatMasker v4.0.5 and Tandem Repeats Finder v4.0.4 (Benson 1999). To detect any additional potential repeats, we partitioned the assembly into 36-mers with an offset of 5 bases, that were mapped back against the reference using GEM v2 (Marco-Sola et al. 2012), allowing a divergence of up to 5%. Any 36-mer with over 20 placements was also masked as a putative repeat undetected by the previous approaches.

We created non-overlapping 36-mers from the raw reads and mapped the 36-mers onto the heavily masked assembly using GEM v2, retaining all possible placements allowing for a divergence of up to 5%. The mappings were then fed to mrCanavar v0.51 (Alkan et al. 2009) to estimate raw copy number in windows of 1 kbp of non-overlapping and non-repetitive sequence, meaning that a given window might span more than 1 kbp in genomic coordinates. To this end we introduced a padding of 36bp to any given region that was masked out in the assembly, to avoid a spurious drop

off in coverage at the boundary of masked sequence that would lead to an underestimation o true copy number. The depth of coverage of any given window was then corrected for GC content, and the absolute copy number calculated as the two times the depth of coverage divided by the median depth of coverage in a set of control windows defined by mrCanavar, which fit the expectation of coverage in single copy regions. For a diploid genome, we expect the copy number values in control regions to form a bell-shaped curve centered at 2. Because of a broad dispersion of these values and an elevated number of windows with high copy number, we excluded the fin whale from subsequent analysis.

Finally, we conservatively called segmental duplications as regions where 5 consecutive windows have a copy number above the mean copy number in control regions plus 3 standard deviations, allowing for one internal window to be above only 2 standard deviations, and required a minimum length of 10 kbp. Additionally, any window with a copy number above 100 was filtered out. To call genes within a segmental duplication, we required the gene annotation to be fully embedded within the duplicated region. Results are summarized in Supplementary Table 6. We visualized intersecting sets of shared versus unique cetacean segmental duplications and the genes annotated on them using UpsetR (Lex et al. 2014).

Clustering of single-copy orthologous genes across mammals

Genomes for 28 mammals were sourced from Ensembl (Zerbino et al. 2018), NCBI (O'Leary et al. 2016) and individual genome sequencing projects (Supplementary Table 10). The entire set of all coding DNA sequences (CDSs) for each species were downloaded and subjected to filters that assessed quality; these included whether the sequences were divisible by three and lacked internal stop codons. Data sourced from Ensembl may contain multiple transcripts for genes; therefore, we applied the "*Clean_Ensembl*" function from VESPA which in addition to the filters mentioned above also filters to these data to retain the longest canonical transcript for every gene (Webb et al. 2017). Only those CDSs that satisfied our quality filters were retained.

There was significant attrition following the application of quality filters for a small number of species, mostly due to incomplete codons in the available gene sets (i.e.

walrus, rhino, and sperm whale, Supplementary Table 11), resulting in the majority of protein families identified lacking representation for these species. However, the sequences retained for further analysis were of high quality due to our filtering, and all major clades were represented in the gene families analyzed. Sequences that passed the quality filters described above were then translated into amino acids and subjected to an all versus all best reciprocal BLASTp (Altschul et al. 1997) with an e-value of 1e-5 and max targets set to 5,000. Single Gene orthologous (SGO) families were identified from the blast output files and both amino acid and the nucleotide files were assembled for each family. In total, 1,152 SGO families were identified.

Using the VESPA package (Webb et al. 2017), we used two methods of alignment for each SGO: (1) MUSCLE (Edgar 2004) and (2) MAFFT (Katoh and Standley 2013), and compared alignments from both methods using the norMD score implemented in the "*metal_compare*" function of VESPA (Blackburne and Whelan 2012). All alignments were carried out at the amino acid level and gaps were placed into the corresponding original nucleotide sequences based on where they occurred in the amino acid sequences.

Rates of molecular evolution and divergence time estimation with r8s

Rates of molecular evolution were estimated on the 4D dataset with the semiparametric penalized likelihood (PL) method implemented in r8s v1.8 (Sanderson 2002; Sanderson 2003). We constrained minimum, maximum or fixed node ages based on the paleontological literature (Benton et al. 2015) (Supplementary Table 9). The PL method estimates a different substitution rate on each branch and implements a penalty when rates differ among branches. This penalty is quantified as a smoothing parameter, which we optimized through cross-validation by allowing values to range on a $\log_{10}$ scale starting from 10^0 with the exponent increasing 0.3 for ten steps. We then reran the analysis with the optimal smoothing parameter value. We also used a gradient check to ensure that active constraints were correct (such as negative if a minimum constraint was used).

Demographic analysis

We used the Pairwise Sequential Markovian Coalescent (PSMC) (Li and Durbin 2011) to reconstruct the population history of North Atlantic humpback whales, including the individual sequenced in the current study (downsampled to ~20X coverage) and a second individual sequenced at ~17X coverage in Árnason et al. (2018). The PSMC infers historical population size changes using the density of heterozygous sites across the genome of a single diploid individual, under the assumption that the rate of coalescent events is inversely proportional to effective population size. We aligned paired-end short insert whole genome shotgun reads to the hardmasked humpback whale assembly using the ‘bwa-mem’ algorithm with bwa v0.7.17 (Li and Durbin 2009). After removing unmapped reads and sorting by position in samtools v1.9 (Li et al. 2009; Li 2011), we marked PCR duplicates using picard-tools v1.125 (http://broadinstitute.github.io/picard/). We then constructed a consensus sequence by generating a .vcf file with ‘mpileup’ in samtools and the bcftools consensus caller. We used vcfutils.pl ‘vcf2fq’ to output a fastq sequence allowing minimum and maximum depths of 0.5 and 2 times the sample sequence depth and a minimum mapping quality of 30. The PSMCs were applied using 64 atomic time intervals (4+25*2+4+6), which included 28 free interval parameters. We performed bootstrapping by splitting the humpback whale scaffolds into shorter segments and running 100 replicates with replacement. As the PSMC results in relative effective population size estimates over time, we rescaled with a mutation rate and generation time. For the mutation rate, we used the pairwise syntenic net of the common minke and humpback whale genome assemblies to calculate the average sequence divergence across all aligned blocks. We then averaged the mean divergence time estimates between common minke and humpback whales across all the methods described above. For an alternate mutation rate, we also plotted the PSMC using the substitution rate for the humpback whale branch that resulted from the penalized likelihood analysis. Following Árnason et al. (2018), PSMCs were scaled assuming a 21.5 year generation time for humpback whales, as estimated for a stable population under pre-disturbance conditions (Taylor et al. 2007).

Non-neutral substitution rates in cetacean genomes

In order to identify genomic regions controlling cetacean-specific adaptations, we used *phyloP* (Pollard et al. 2010) to detect loci in the 12-mammal WGA that depart from neutral expectations. Given a null distribution for the total number of substitutions at a locus under a nonconserved evolutionary model, *phyloP* will estimate the observed number of substitutions in the alignment and compute a P value by comparing this estimate to the null distribution using a likelihood ratio test (LRT). We used the 4D sites from the WGA as a neutral model of evolution, fit a time-reversible substitution model to the best ML phylogeny and estimated branch lengths in terms of substitutions per site using *phyloFit* in PHAST. To determine which protein-coding genes contain cetacean-specific accelerated regions, we first assessed 10bp windows of the alignment that departed from the neutral model in the cetacean subtree, using the LRT and a significance cutoff of 3.1E-9 in order to account for the large number of sites in the alignment and to avoid false positives (Pollard et al. 2010). We then collected accelerated regions that overlapped human whole gene annotations (hg19) using *bedtools intersect* (Quinlan and Hall 2010) and tested for the enrichment of Gene Ontology (GO) terms using the PANTHER analysis tool available at the Gene Ontology Consortium website (Gene Ontology database, last accessed June 2017) (Gene Ontology Consortium 2015).

Detection of protein-coding genes subjected to positive selection

We used codon-based models to test for selective pressure variation along branches of the cetacean phylogeny in comparison to other mammal using PAML v4.4e (Yang 2007). The following 5 branches were assessed as foreground using the branch-site models of evolution described below: humpback whale; the most recent common ancestor (MRCA) of the common minke and humpback, MRCA of baleen whales; MRCA of toothed whales, and the MRCA of all whales (cetacean stem lineage).The models used for this analysis allow for heterogeneous rate ratios of nonsynonymous substitutions per non-synonymous site (d_N) to synonymous substitutions per synonymous site (d_S) or ($\omega = d_N/d_S$) across sites and amongst branches/lineages. An ω-value > 1 indicates positive selection, $\omega < 1$, purifying selection and neutral evolution

when $\omega = 1$. The statistically significant model for the data was selected using a series of likelihood ratio tests (LRTs) to compare models and their more parameter rich extensions. Both lineage-specific models and site-specific models were evaluated using LRT. Sequences were considered to exhibit lineage-specific selective pressure if the LRT for ModelA was significant in comparison to both ModelA null and M1Neutral, where M1Neutral is a neutral model that allows two site classes: ω_0=0 and ω_1=1. Model A assumes the two site classes are the same in both foreground and background lineages (ω_0=0 and ω_1=1) and ω_2 was calculated from the data. Model A null was the null hypothesis for this model and allowed sites to be either subjected to purifying selection or to be neutrally evolving in the background lineages. For site-specific analyses, LRTs were conducted to compare models M7 and M8a with model M8. The test compared the neutral model M7, which assumes a β distribution for ω over sites and the alternative model M8 (β and ω), which adds an extra site class of positive selection. M8a is the null hypothesis of M8 where the additional category is neutral, i.e. ω=1. Codon frequencies were estimated empirically from the data and were calculated using likelihood and based on the base frequencies (codon freq =2 F3x4 in the codeml control file). We ran each likelihood model with 4 different starting omega values (0,1,2, and 10) and reported our estimates of the parameters (proportion of sites, omega values and likelihood values and sites identified as selected under BEB) from the lowest likelihood value obtained. This was to ensure we were not reporting our LnL values and associated parameters of interest from a local minimum.

The VESPA functions "*codeml_setup*" and "*codeml_reader*" are automated CodeML wrappers and were used to prepare all the codeML files, to parse the PAML output and perform the likelihood ratio tests (Webb et al. 2017). The probability (PP) of a specific amino acid site belonging to the positively selected category is estimated using the empirical Bayes method for each superfamily individually. After ML estimates of model parameters were obtained, we used two bayesian approaches to infer the posterior probability of the positively selected sites: Bayes Empirical Bayes (BEB) and Naïve Empirical Bayes (NEB) (Yang and Nielsen 2002). BEB reduces the rate of false positives when analyzing small datasets and retains the power of NEB when analyzing

large datasets. Therefore, if NEB and BEB were both predicted the results from BEB were preferred.

To minimize the impact of potential false positives the putative positively selected sites were manually inspected. Using VESPA-produced alignments, the putative positively selected sites were removed if: i) the aligned position of the positively selected site was in question - i.e. proximal or within a poorly conserved section of the alignment, ii) the positively selected site produced a substitution found at that position in other species not encompassed by the foreground label - i.e. other organisms within the alignment also possess the reported substitution at that position, iii) less than seven species at the position of the putative positively selected site.

TP53 cloning and sequencing

Primers were designed based upon scaffold ScjyzU5_270, positions 2002892 to 2005157) using PRIMER3 (ver. 0.4.0 Koressaar and Remm, 2007; Untergasser et al., 2012) aiming at an annealing temperature at 57 °C. Th eproposed primers were doubled checked using AMPLIFX (ver.1.7.0 Nicolas Jullien ; CNRS, Aix-Marseille Université - https://inp.univ-amu.fr/en/amplifx-manage-test-and-design-your-primers-for-pcr").

Table 1. Primer sequences.

Primer	5'position in scaffold ScjyzU5_270	Primer sequence (5'-3')
TP53aR	2005039	ACG TTC ATC CAG CCA GTT TG
TP53bF	2004576	GTA TGT CTC ACG CTG GAT CCT C
TP53bR	2004452	TCA CCA CGC AGA GGG ACT TCC A
TP53aF	2003908	GCT GGT CTG AGA GAT GAG AT

Notes: F and R denotes forward and reverse oriented primer relative to the scaffold sequence.

Figure 1. Relative position of PCR primers

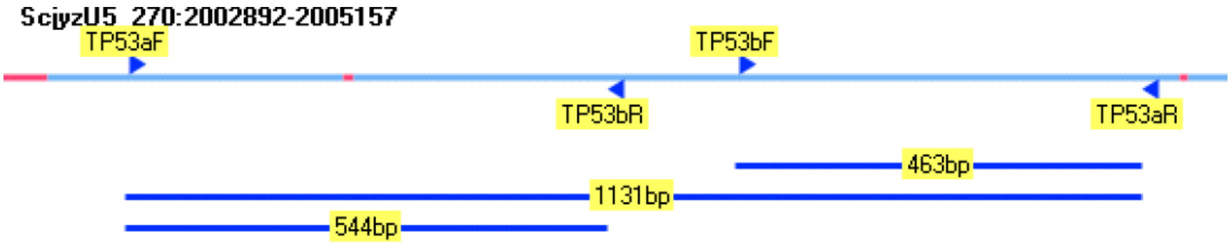

A fragment of 1131 bps of TP53 were amplified using the primers TP53aF and TP53aR (Table 1). The initial PCR amplifications were performed in a 10 μL volume reaction consisting of 0.2 μM of each dNTP, 5x Phusion™ high fidelity buffer (NEB Inc.), 0.1μM of each primer, 0.4 units of Phusion™ *Taq* DNA polymerase (NEW Inc.) and ~ 10 -20 ng of DNA extraction. The thermo-cycler conditions were: 2 min at 98 °C, followed by 35 cycles at 98 °C (30 sec), 60 °C (30 sec) and 72 °C (60 sec) and a final single step cycle at 72 °C (10min). The quality of the amplification products was assessed by gel electrophoresis in 2% agarose in 1xTBE at 175 volts for 35 minutes. PCR products were cloned using the Zero® Blunt Topo® PCR Cloning kit (Life Technologies Inc.) following the manufacturer's instruction and a vector to fragment ratio of 1:2. Positive colonies were collected and eluted in 0.1xTE buffer heated to 98 ºC for 8 minutes.

Two fragments of 544 bps (primers TP53aF and TP53bR) and 463 bps (TP53bF and TP53aR) were amplified and sequenced from each clone. PCR amplifications were performed as described above, except the Phusion Taq DNA polymerase and buffer were replaced a standard *Taq* DNA polymerase and buffer (Life Technologies Inc.) and the PCR product was assessed as described above. PCR amplifications yielding a single PCR product of the expected size were sequenced (as described above).
A total of 26 (of 96) clones yielded unambiguous DNA sequences of both the 544 and 463 bp fragment, which aligned to the TP53 sequence identified in scaffold ScjyzU5_270, positions 2002892 to 2005157. In total two SNPs were detected; in positions 1237 and 1875 (Table 2).

Table 2. Haplotypes of the 26 clones with unambiguous DNA sequences.

Clones	Position 1237 "G/A"	Position 1875 "C/G"	Tp53 haplotype
Clone1	A	G	Tp53-1
Clone2	A	C	Tp53-2
Clone3	G	C	Tp53-3
Clone18	A	C	Tp53-2
Clone19	G	G	Tp53-4
Clone21	G	C	Tp53-3
Clone22	G	C	Tp53-3
Clone23	G	C	Tp53-3
Clone24	A	G	Tp53-1
Clone25	G	G	Tp53-4
Clone32	G	G	Tp53-4
Clone36	A	G	Tp53-1
Clone38	A	C	Tp53-2
Clone41	G	G	Tp53-4
Clone46	G	C	Tp53-3
Clone48	G	G	Tp53-4
Clone49	A	C	Tp53-2
Clone51	A	C	Tp53-4
Clone52	G	G	Tp53-4
Clone57	A	G	Tp53-1
Clone58	A	G	Tp53-1
Clone67	A	G	Tp53-1
Clone68	A	C	Tp53-2
Clone81	G	C	Tp53-3
Clone89	A	C	Tp53-2
Clone93	A	C	Tp53-2

Table 3. Haplotype frequencies

Haplotype number	Count	Clone/sequence
Tp53-1	6	Clone1, Clone24, Clone36, Clone57, Clone58, Clone67
Tp53-2	8	Clone18, Clone2, Clone38, Clone49, Clone51, Clone68, Clone89, Clone93
Tp53-3	7	Clone21, Clone22, Clone23, Clone3, Clone46, Clone81, ScjyzU5_270:2002892-2005157
Tp53-4	6	Clone19, Clone25, Clone32, Clone41, Clone48, Clone52

Supplementary Figure 1. Cumulative distribution frequencies (CDF) of Annotation Editing Distances (AED) for four humpback whale genome annotations using different ab initio gene predictors. All annotations included aligned evidence from skin transcriptome, nine mammalian proteomes and the Swiss-Prot database.

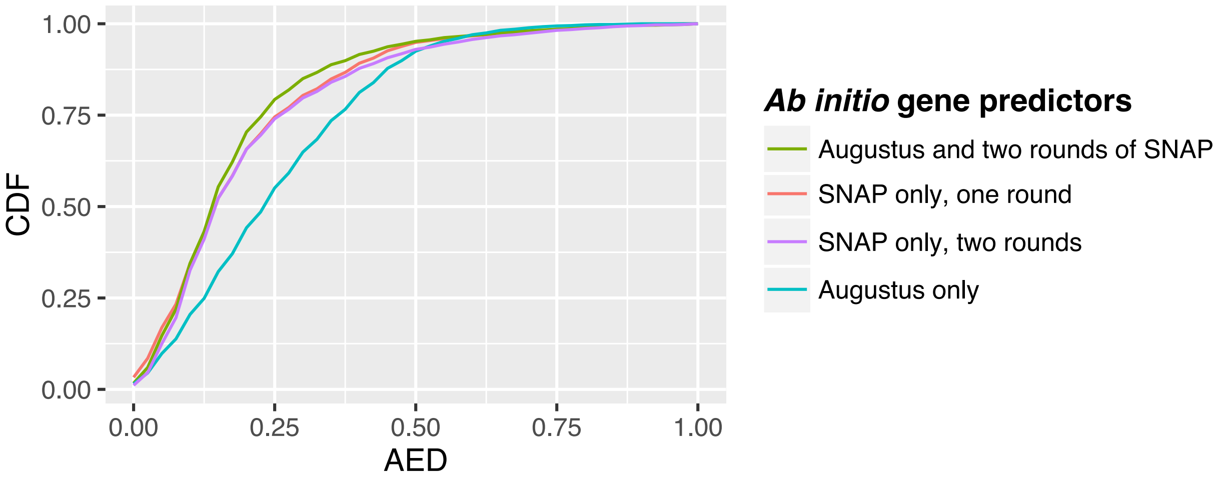

Supplementary Figure 2. Repeat landscapes for the humpback whale genome derived from *de novo* (RepeatModeler) and database (RepBase) libraries.

Humpback whale (*Megaptera novaeangliae*)

RepeatModeler:

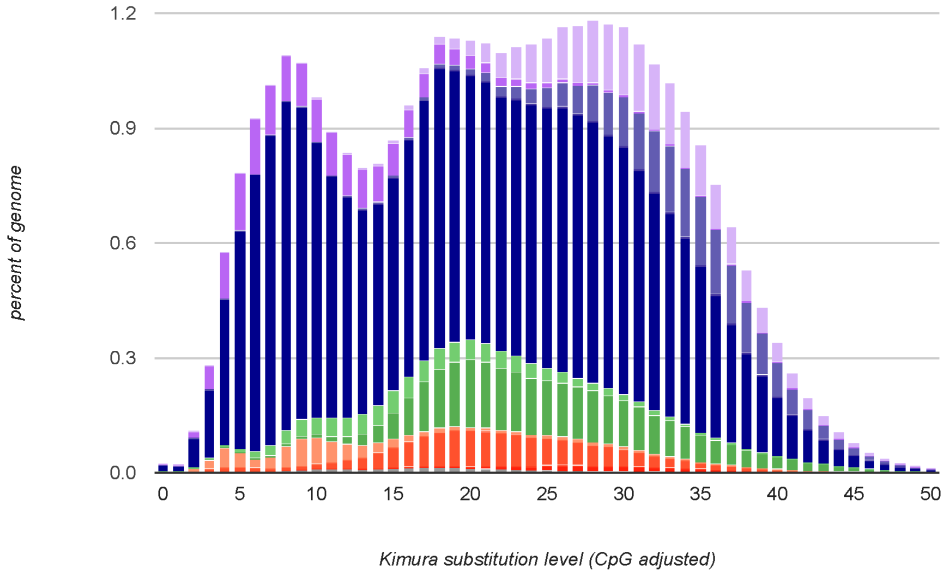

RepBase:

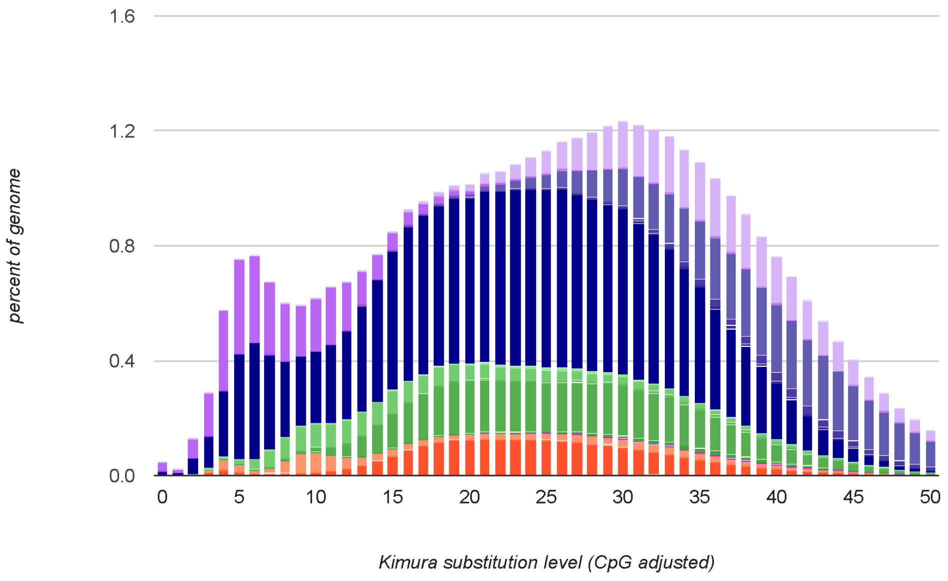

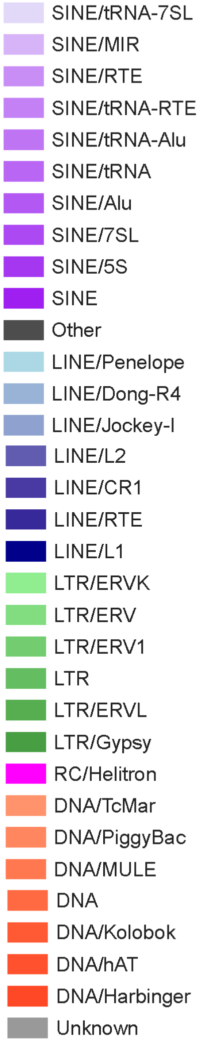

Supplementary Figure 3. Repeat landscape for the bowhead whale genome derived from the RepBase library.

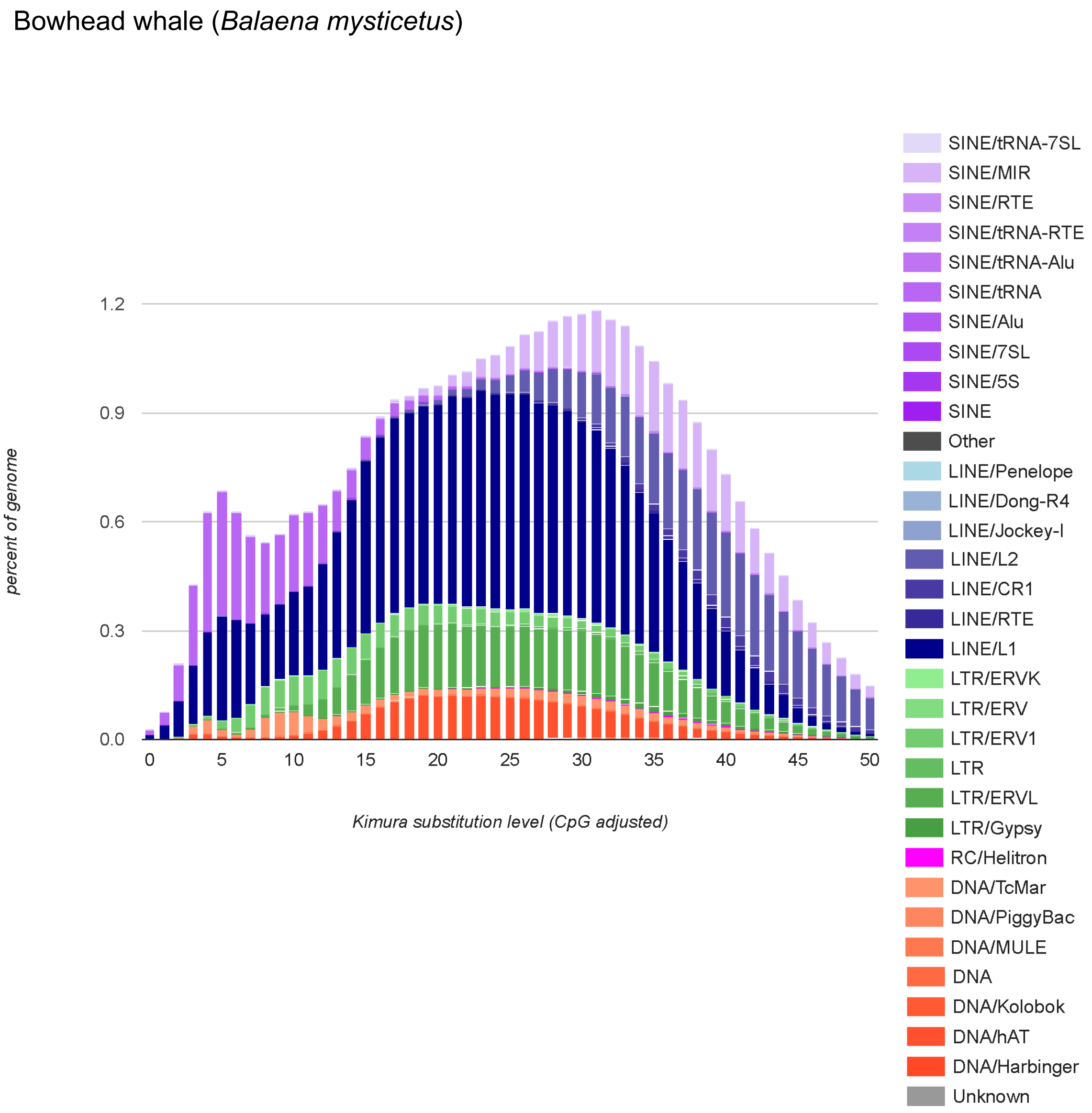

Supplementary Figure 4. Species tree phylogeny using 152 maximum likelihood gene trees with ASTRAL. Local posterior probabilities are given for each branch. Branch lengths are in terms of coalescent units and measure gene tree discordance.

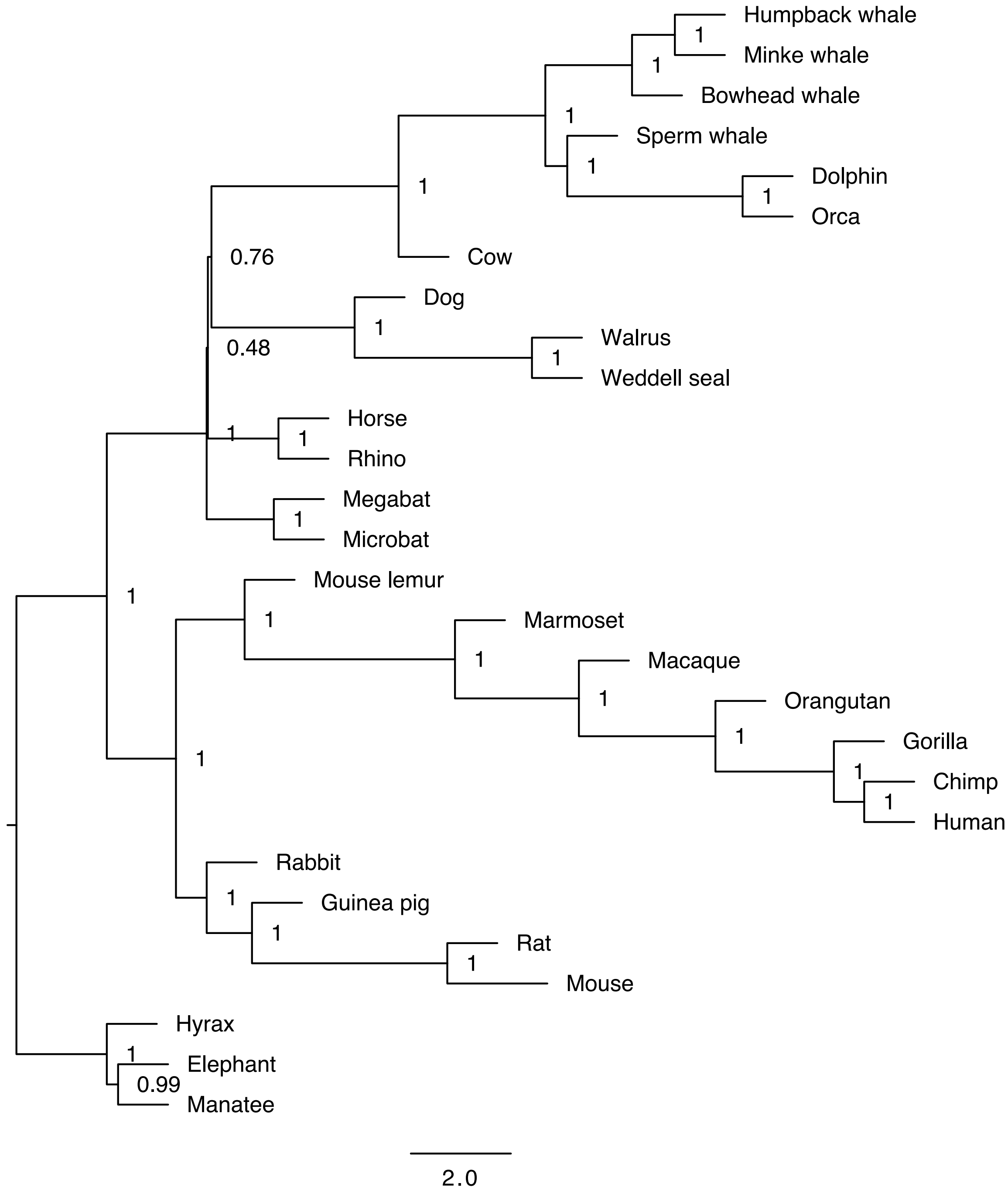

Supplementary Figure 5. Phylogeny with node labels used for the MCMCtree and r8s analyses using fourfold degenerate site data.

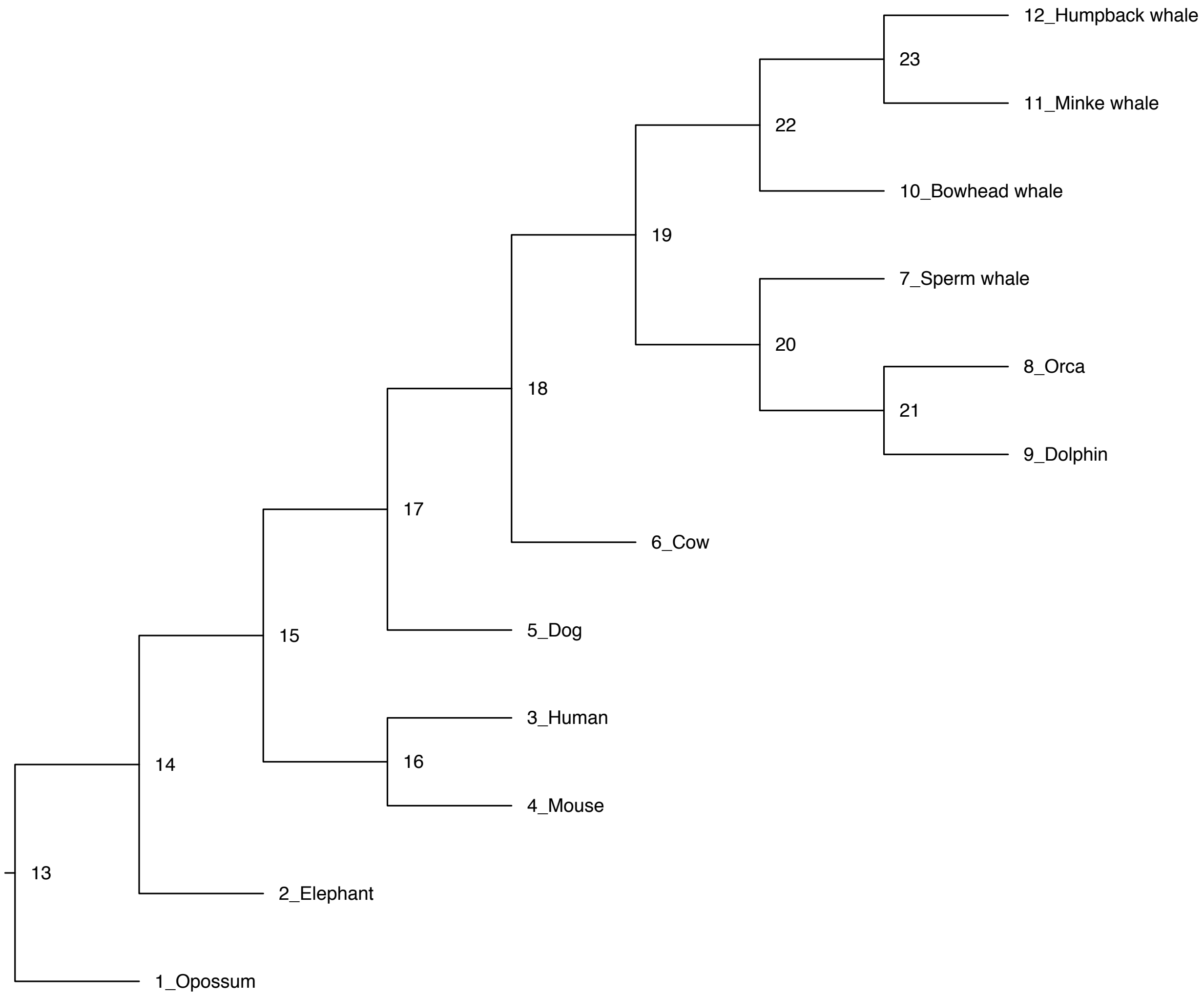

Supplementary Figure 6. Phylogeny with node labels used for the MCMCtree analysis using 152 single-copy orthologs.

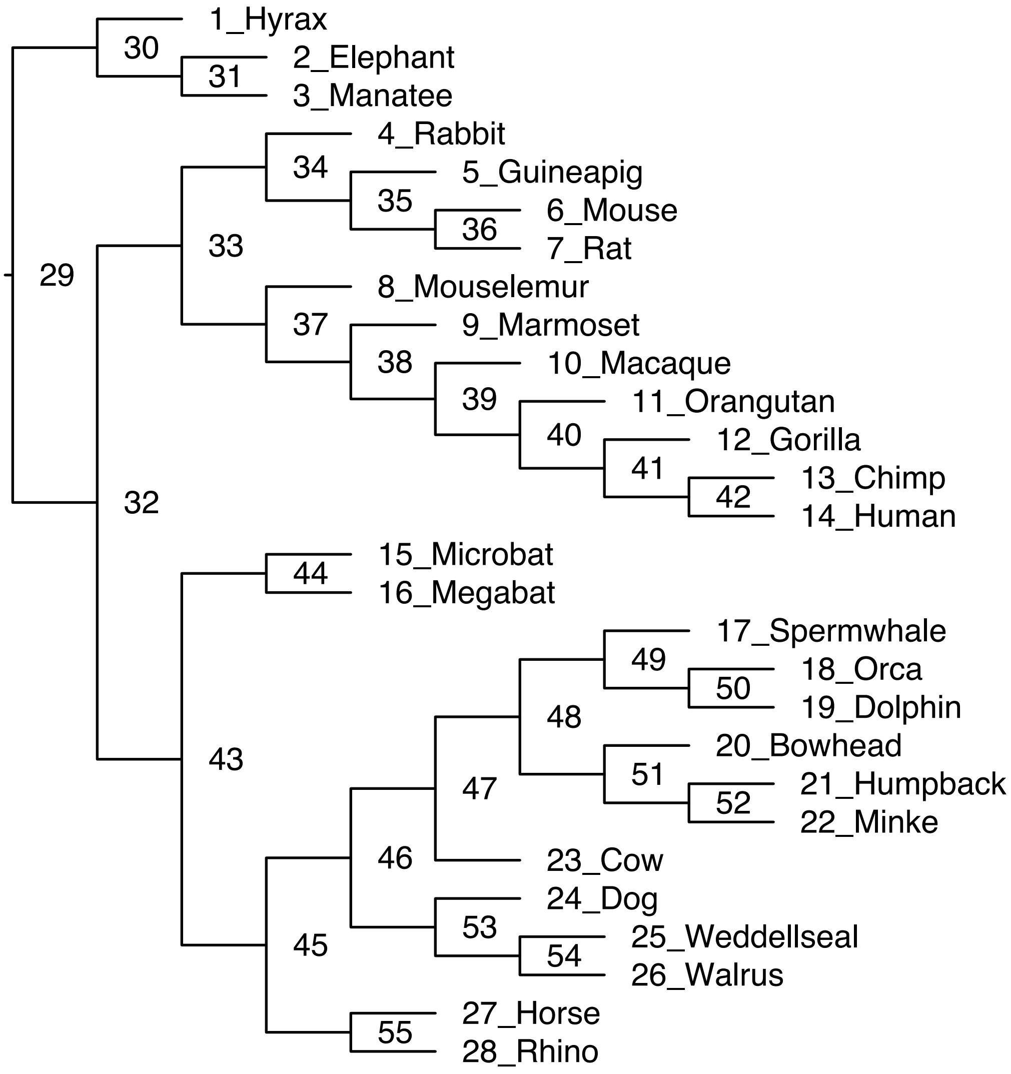

Supplementary Figure 7. Pairwise Sequential Markovian Coalescent (PSMC) analyses with 100 bootstraps for two North Atlantic humpback whales. Mutation rate was 2.2e-9, derived from the penalized likelihood analysis. (A) Salt, the whale sequenced for the current study; (B) 17X coverage data for a humpback whale from Arnason et al. (2018).

A

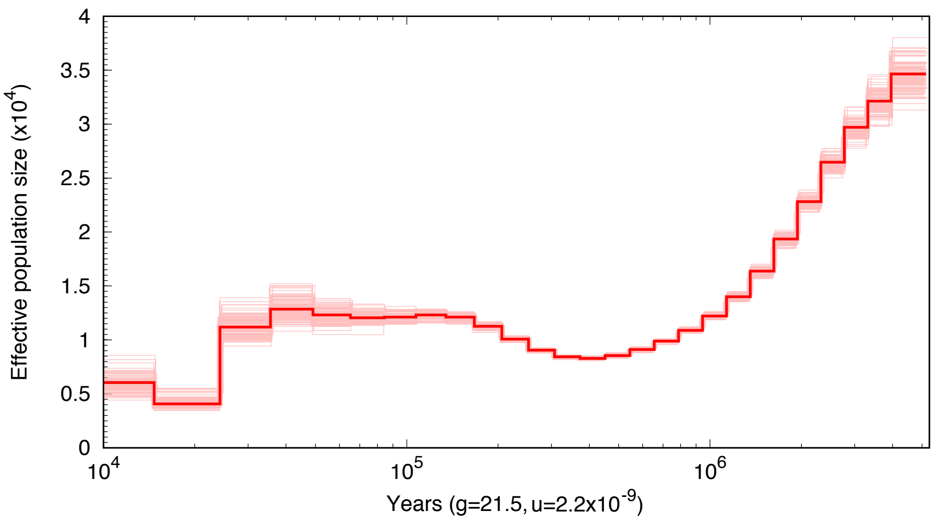

B

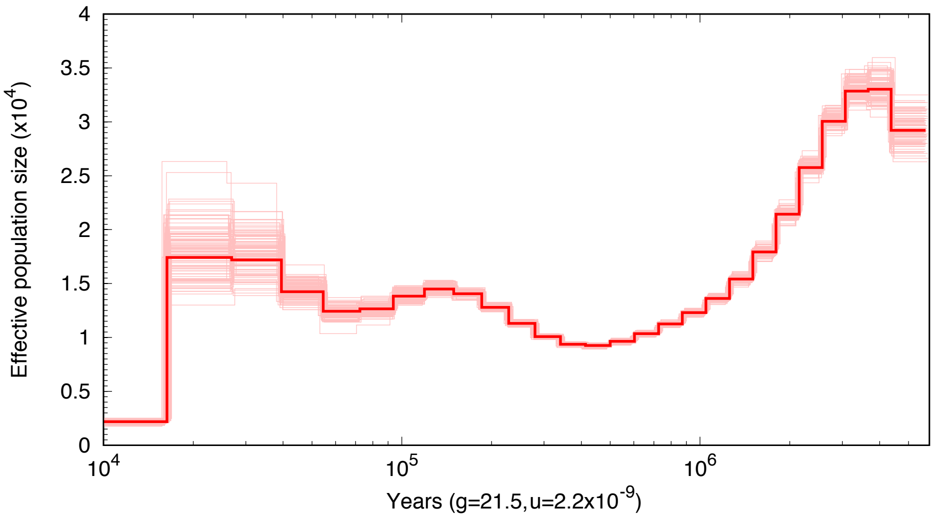

Supplementary Figure 8. Pairwise Sequential Markovian Coalescent (PSMC) analyses with 100 bootstraps for two North Atlantic humpback whales. Mutation rate was 1.54e-9, derived from both genomic sequence divergence and divergence time estimates of minke and humpback whales. (A) Salt, the whale sequenced for the current study; (B) 17X coverage data for a humpback whale from Arnason et al. (2018).

A

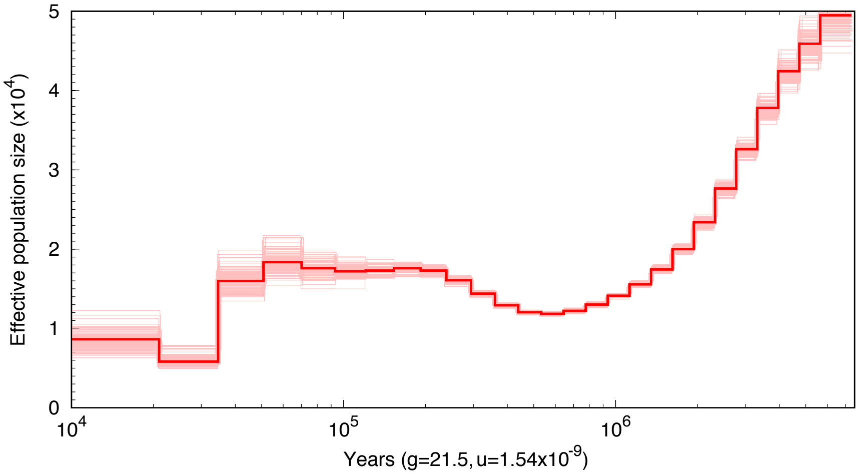

B

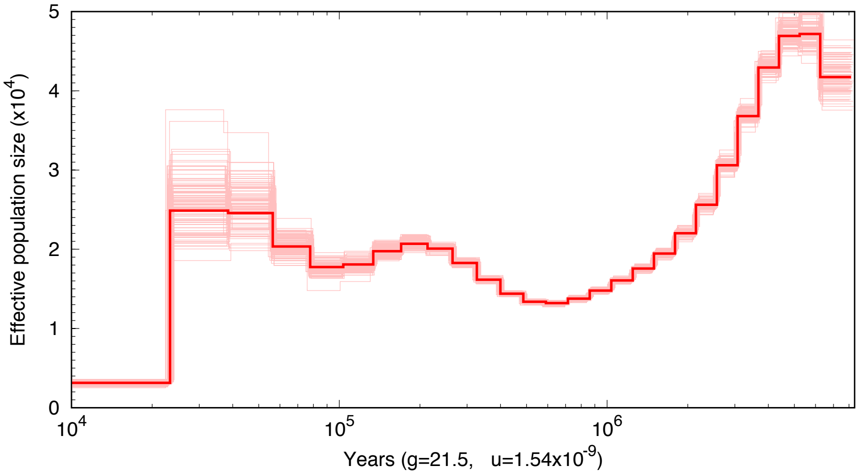

Supplementary Figure 9. Intersecting sets of (A) large segmental duplications and (B) genes annotated on large segmental duplications across 11 cetacean genomes.

A

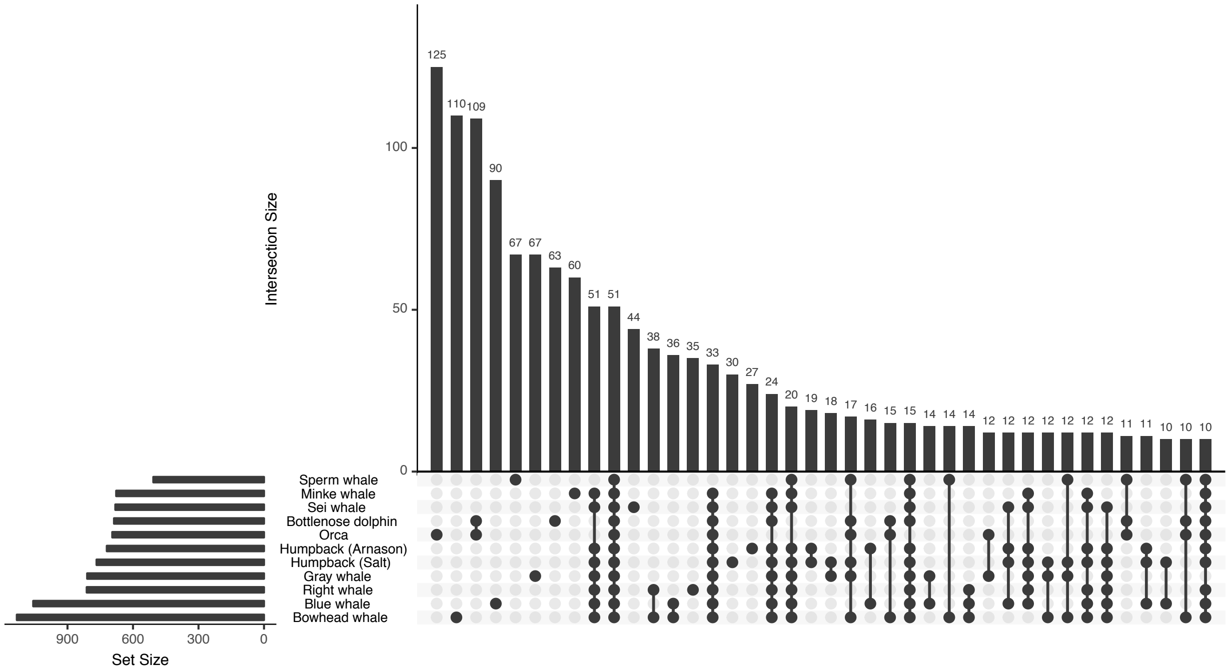

B

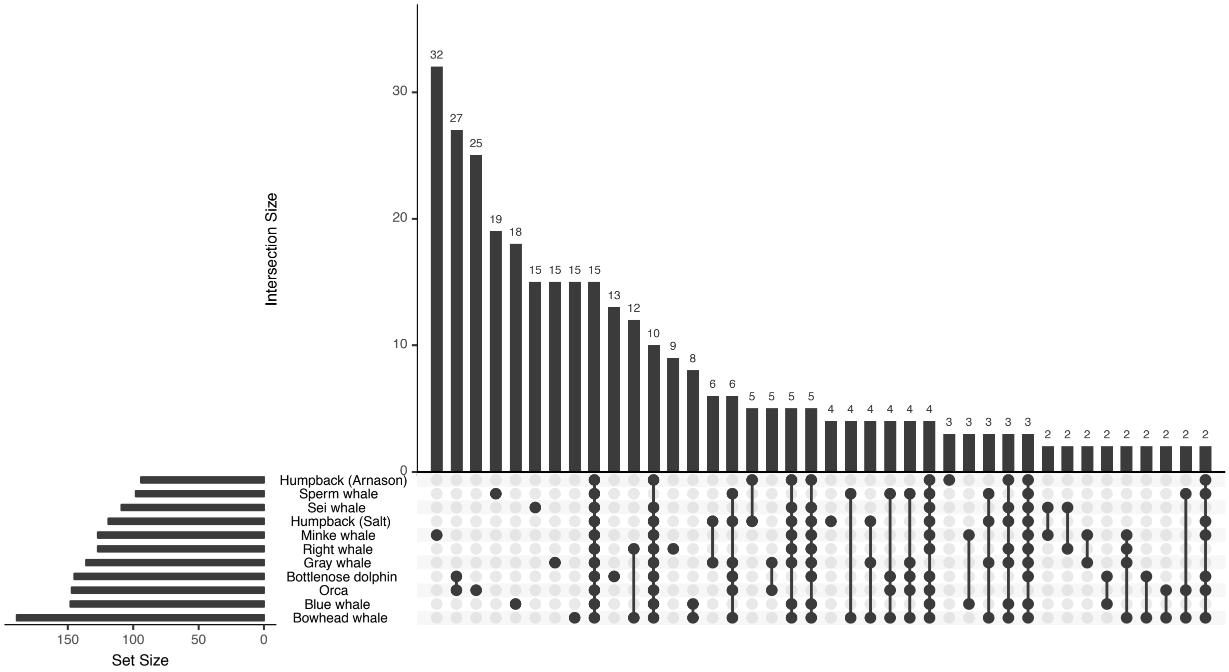

Supplementary Figure 10. Protein interaction networks of positively selected genes unique to the humpback whale.

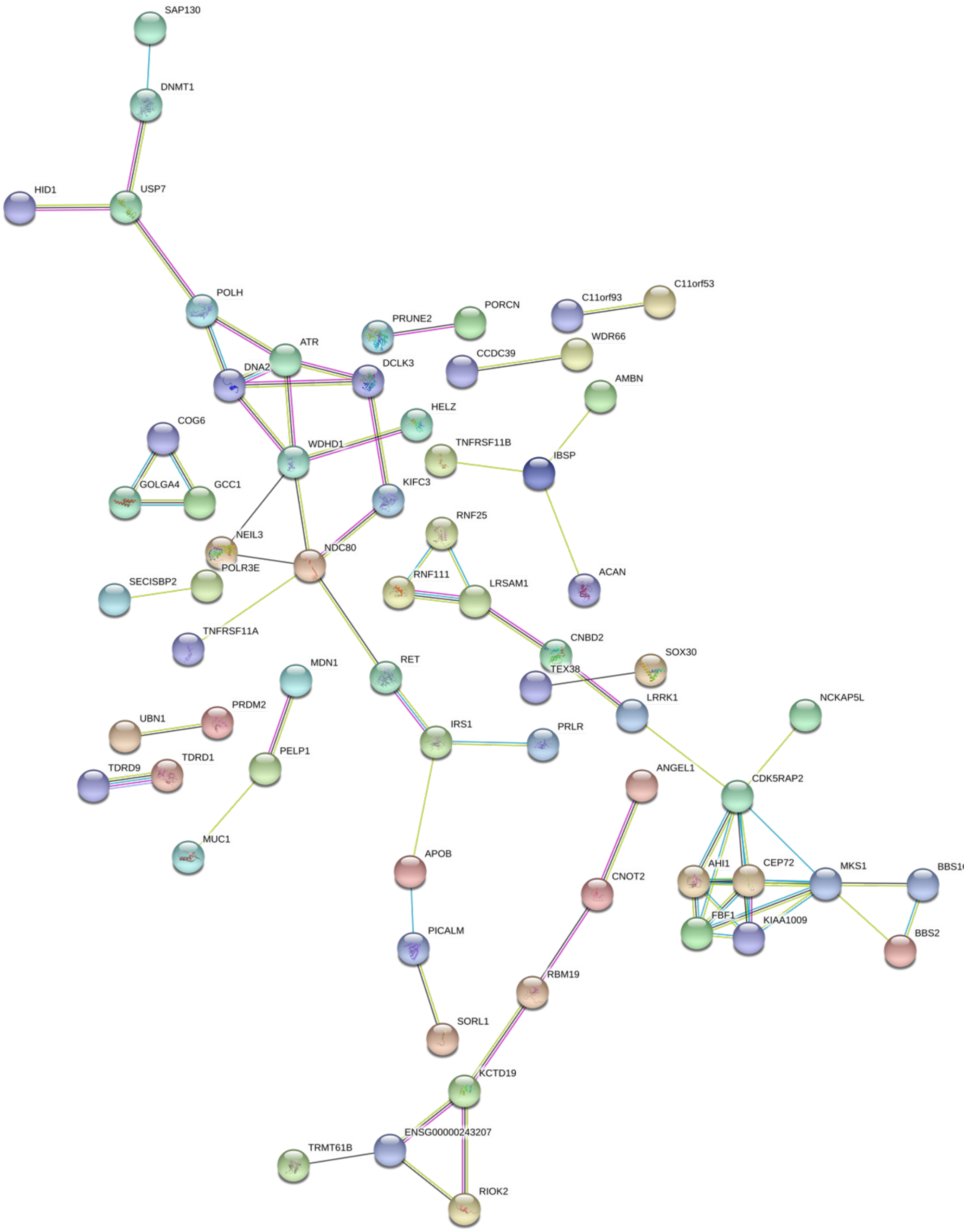

Supplementary Table 1. Summary of gene annotations in six mammalian genomes.

**Assembly**	megNov1		turTru2[1]		bosTau7[1]		hg38[1]		mm10[1]		loxAfr3[1]	
**Species name**	*Megaptera novaeangliae*		*Tursiops truncatus*		*Bos taurus*		*Homo sapiens*		*Mus musculus*		*Loxodonta africana*	
**Total number of protein coding genes**	24,140		16,550		19,981		19,923		22,064		20,033	
	**Average**	**Median**	**Average**	**Median**	**Average**	**Median**	**Average**	**Median**	**Average**	**Median**	**Average**	**Median**
**Gene length**	22,691	10,353	36,802	10,339	32,882	9,269	47,276	10,671	34,376	7,225	33,637	8,241
**Exons per mRNA**	8	5	13	8	10	6	13	4	12	4	10	6
**Exon length**	194	131	125	106	203	127	250	129	285	132	156	119
**Intron length**	2,828	1,102	1,690	672	4,705	1,154	6,670	1,604	5,405	1,363	3,905	1,127

[1]Obtained from Ensembl version 92.

Supplementary Table 2. Genome assemblies used in the whole genome alignment.

**Species**	**Genome Assembly**
Human	hg19*
Mouse	mm10*
elephant	loxAfr3.0*
opossum	monDon5.0*
Dog	canFam3.0*
Cow	bosTau7*
Minke whale	balAcu1*
Bottlenose dolphin	turTru2*
Orca	GCA_000331955.2 Oorc_1.1
Sperm whale	GCA_002837175.1 ASM283717v1
Bowhead whale	v1.0
Humpback whale	Current study

*Downloaded from UCSC Genome Browser

Supplementary Table 3. Substitution rate and divergence time estimates from the penalized likelihood analysis in r8s on fourfold degenerate site data, using node labels from Supplementary Figure 5.

Clade/Tip Name	Node	Age (MY)	Estimated Rate
Theria	13	164*	--
Opossum	1	-	2.2225e-03
Eutheria	14	87.39	2.2467e-03
Elephant	2	--	2.2423e-03
Boreoeutheria	15	80.05	2.2477e-03
Euarchontoglires	16	73.58	2.2564e-03
Human	3	--	2.2521e-03
Mouse	4	--	2.2688e-03
Laurasiatheria	17	70.54	2.2306e-03
Dog	5	--	2.2375e-03
Cetartiodactyla	18	52.40	2.2300e-03
Cow	6	--	2.2304e-03
Cetacea	19	33.9	2.2196e-03
Odontocetes	20	19.59	2.2141e-03
Sperm whale	7	--	2.2125e-03
Delphinidae	21	4.33	2.2125e-03
Orca	8	--	2.2123e-03
Dolphin	9	--	2.2125e-03
Mysticetes	22	11.19	2.2144e-03
Bowhead whale	10	--	2.2133e-03
Rorquals	23	7.57	2.2137e-03
Humpback whale	12	--	2.2138e-03
Minke whale	11	--	2.2132e-03

MY=million years; *fixed node age.

Supplementary Table 4. Divergence time estimates from the MCMCtree analysis using the fourfold degenerate site data.

Node*	Mean (MY)	95% HPD interval (MY)
13	163.3783	[156.4333, 169.7255]
14	106.9612	[99.687, 111.5559]
15	93.7316	[83.1746, 104.3303]
16	76.3801	[61.03, 91.6215]
17	79.2541	[66.9006, 92.0377]
18	58.1154	[52.0391, 65.2754]
19	35.4608	[31.4781, 40.9357]
20	30.7658	[22.4024, 38.5593]
21	7.3567	[1.8082, 15.1848]
22	20.4864	[9.1455, 31.8335]
23	13.7549	[4.1291, 24.8486]
mu1	1.801E-3	[1.2836E-3, 2.4506E-3]

*Node labels correspond to Figure S5. MY = million years. HPD = highest posterior density. mu1 = substitution rate.

Supplementary Table 5. Divergence time estimates from the MCMCtree analysis using single-copy orthologs with node labels corresponding to Supplementary Figure 6.

Node*	Mean (MY)	95% HPD interval (MY)
t_n29	110.79	[107.5075, 113.9817]
t_n30	63.3264	[53.7549, 72.5819]
t_n31	56.6599	[46.8725, 65.8416]
t_n32	109.3561	[104.7489, 112.896]
t_n33	99.2497	[94.3413, 103.7053]
t_n34	89.9237	[84.7833, 95.2143]
t_n35	75.1066	[69.0466, 81.5027]
t_n36	30.6078	[24.2418, 36.6735]
t_n37	86.0007	[79.2763, 92.0792]
t_n38	38.6887	[33.6413, 44.4188]
t_n39	25.0177	[23.5929, 27.1406]
t_n40	13.6617	[11.1849, 16.1283]
t_n41	6.5398	[5.2576, 7.8834]
t_n42	4.8686	[3.7285, 6.1542]
t_n43	92.7646	[87.0903, 98.1149]
t_n44	72.6762	[64.3949, 80.8283]
t_n45	89.8759	[84.1297, 95.074]
t_n46	85.4227	[79.7001, 90.6196]
t_n47	56.7765	[51.8901, 62.504]
t_n48	32.2635	[28.7514, 35.1176]
t_n49	29.727	[26.2037, 32.7341]
t_n50	6.4951	[4.7164, 8.4037]
t_n51	22.1972	[18.4651, 25.8003]
t_n52	18.1005	[14.6431, 22.0267]
t_n53	52.6333	[43.892, 61.1608]
t_n54	20.8068	[15.4274, 26.4335]
t_n55	70.6363	[61.2855, 79.2097]
mu1	7.77E-04	[6.99E-4, 8.554E-4]
mu2	6.62E-04	[6.012E-4, 7.267E-4]
mu3	1.66E-03	[1.4718E-3, 1.8632E-3]

* MY = million years. HPD = highest posterior density. mu1, mu2, mu3 = substitution rates for 1st, 2nd, and 3rd codon positions, respectively.

Supplementary Table 6. Summary of Large Segmental Duplication (LSD) Analysis in Cetacean Genomes.

Species	Short Read Archive Accession #	Production bases used	# LSDs	length LSDs (bp)	LSD length (bp)	Largest LSD (bp)	# genes in LSDs	# of quality in LSDs[1]
Humpback whale (Salt)	Current study	292,629,498,475	293	10,128,534	34,568	364,308	119	28
Humpback whale (Arnason)	SRR5665639	52,464,651,300	283	8,166,656	28,857	285,539	95	19
Blue whale	SRR5665644	113,023,546,414	379	12,523,750	33,044	888,626	148	36
Minke whale	SRR5665640	52,464,651,300	291	10,115,414	34,761	804,460	127	43
Sei whale	SRR5665645	33,452,041,860	279	9,089,414	32,579	531,454	109	28
Gray whale	SRR5665641	52,464,651,300	338	10,178,957	30,115	405,593	136	29
Bowhead whale	SRR1685383	14,908,354,6518	432	12,839,657	29,721	159,672	189	50
North Atlantic right whale	SRR5665640	33,477,861,780	289	8,557,321	29,610	202,443	127	28
Sperm whale	SRR5665645	37,399,955,800	235	6,768,750	28,803	146,736	98	30
Orca	SRR574978<br>SRR574981	79,971,263,892	367	10,282,776	28,019	297,699	147	43
Bottlenose dolphin	SRR606319<br>SRR606320<br>SRR606321<br>SRR606322<br>SRR606323	90,953,286,400	313	9,889,052	31,594	297,699	145	39

[1]Genes with one-to-one orthology to human gene set (Ensembl)

Supplementary Table 7. Summary of Large Segmental Duplications (LSDs) that are unique to each cetacean genome.

Species	Number of unique LSDs	Total length of unique LSDs (bp)	Average length of unique LSDs (bp)
Blue whale	90	2,370,100	26,334
North Atlantic right whale	35	443,370	12,668
Bottlenose dolphin	63	920,330	14,608
Gray whale	67	1,138,060	16,986
Minke whale	60	3,553,772	59,230
Sei whale	44	1,956,942	44,476
Bowhead whale	110	1,538,167	13,983
Orca	125	1,950,020	15,600
Sperm whale	67	1,684,405	21,907
Humpback whale (current study)	30	657,214	21,907
Humpback whale (Arnason et al. 2018)	27	319,686	11,840
Merged humpback whale	57	976,900	33,747
Average all species	65	1,459,081	24,441

Supplementary Table 8. Genes from the COSMIC database evolving under accelerated evolution in pairwise genomic comparisons.

Comparison	Gene Symbol
Minke-humpback	AKR1B10
	CD274
	EAF2
	EDN2
	ETNK1
	GGNBP2
	IGFBPL1
	IL21R
	LETMD1
	MBD2
	MYOD1
	NIT2
	PF4
	PHF6
	PTH1R
	STARD8
	TMPRSS11A
	UPP1
Orca-dolphin	BHLHE41
	BTG1
	CCDC136
	CD274
	CD58
	CXCL12
	E2F5
	FANCD2
	FAS
	FGFR4
	GALR1
	GPC3
	HLTF
	HOXD11

	HOXD13
	KLK10
	LASP1
	LTF
	MLF1
	MYB
	MYD88
	NOX4
	NR4A3
	PALB2
	PML
	RAD21
	RASSF1
	RASSF1
	SAPCD2
	STIL
	TAL1
	TNFRSF10A
	TNFRSF10B
	TNFRSF14
	TNFRSF17
	TNFSF10
	UBE2I
	UPP1
	VTCN1
	YWHAQ
	ZMYND10

Supplementary Table 9: Species included in our analysis are shown along with the genome versions and the number of overall sequences present after filtering.

**Species**	Genome Version used: **Ensembl**/*NCBI project*/Other	**number of seqs**	**Number of sequences following Quality filters**
human	**GRCh38.p7**	215929	22414
chimp	**CHIMP2.1.4**	29160	18740
gorilla	**gorGor3.1**	35727	20940
orangutan	**PPYG2**	29447	20410
macaque	**Mmul_8.0.1**	56748	21073
marmoset	**C_jacchus3.2.1**	55116	20971
mouse lemur	**Mmur_2.0**	43492	18085
mouse	**GRCm38.p4**	119745	22312
rat	**Rnor_6.0**	40459	22239
guinea pig	**cavPor3**	26129	18661
rabbit	**OryCun2.0**	24964	19281
dog	**CanFam3.1**	39074	19841
weddell's seal	*LepWed1.0*	27755	5998
walrus	*Oros_1.0*	33455	5506
horse	**EquCab2**	29196	20384
rhino	*CerSimSim1.0*	38035	2174
megabat	**pteVam1**	22257	16977
microbat	**Myoluc2.0**	26840	19722

cow	**UMD3.1**	26740	19970
orca	_*GCA-000331955.2*	26503	26503
Bottlenose dolphin	**turTru1**	21326	16524
sperm whale	*Physeter_macrocephalus-2.0.2*	36572	4263
minke	*GCA-000493695.1*	32725	32725
bowhead	*PRJNA194091*	19879	19879
humpback	megNov1	24410	15650
elephant	**loxAfr3**	28847	20020
hyrax	**proCap1**	18954	16036
manatee	*TriManLat1.0*	27642	11763

Supplementary Table 10. Node constraints used for divergence time and substitution rate analyses.

Node	Minimum Constraint (MY)	Maximum Constraint (MY)	Citation
Therian ancestorϕ	156.3	164.6	Benton (2015)
Eutherian ancestorϕ	89	111	Hedges et al. (2015)
Eutherian ancestorλ	61.6	164.6	Benton (2015)
Afrotherian ancestor*	56	164.6	Benton (2015)
Euarchontoglires ancestorϕ	61.6	164.6	Benton (2015)
Catarrhine ancestor*	24	34	Benton (2015)
Great ape ancestor*	11.6	33.9	Benton (2015)
Laurasiatherian ancestorϕ	61.6	164.6	Benton (2015)
Cetartiodactyl ancestorϕ	52.4	66	Benton (2015)
Cetacean ancestorϕ	33.9	56	Benton (2015)
Mysticetes ancestorϕ	–	28	Mitchell et al. (1989)

ϕUsed for both fourfold degenerate site and single-copy ortholog datasets *Used in analysis of single-copy orthologs only. λUsed in r8s analysis only. MY = million years.

Supplementary Table 11: Attrition numbers for the walrus, rhino, and sperm whale gene sets.

Species	Total CDS	Filtering Mechanism		Total Removed	Total Passed
		Incomplete Codon	Internal Stop Codon		
Walrus	33,455	18,667	9,282	27,949	5,506
Rhino	38,035	23,882	11,979	35,861	2,174
Sperm whale	36,527	22,370	9,894	32,264	4,263

Supplementary References

Altschul SF, Madden TL, Schaffer AA, Zhang J, Zhang Z, Miller W, Lipman DJ. 1997. Gapped BLAST and PSI-BLAST: a new generation of protein database search programs. Nucleic Acids Res. 25:3389–3402.

Árnason Ú, Lammers F, Kumar V, Nilsson MA, Janke A. 2018. Whole-genome sequencing of the blue whale and other rorquals finds signatures for introgressive gene flow. Sci Adv. 4:eaap9873.

Benson G. 1999. Tandem repeats finder: a program to analyze DNA sequences. Nucleic Acids Res. 27:573–580.

Benton MJ, Donoghue PCJ, Asher RJ, Friedman M, Near TJ, Vinther J. 2015. Constraints on the timescale of animal evolutionary history. Palaeontol Electron 18:1–106.

Blackburne BP, Whelan S. 2012. Measuring the distance between multiple sequence alignments. Bioinformatics 28:495–502.

Edgar RC. 2004. MUSCLE: multiple sequence alignment with high accuracy and high throughput. Nucleic Acids Res. 32:1792–1797.

Eilbeck K, Moore B, Holt C, Yandell M. 2009. Quantitative measures for the management and comparison of annotated genomes. BMC Bioinformatics 10:67.

Foote AD, Liu Y, Thomas GWC, Vinař T, Alföldi J, Deng J, Dugan S, van Elk CE, Hunter ME, Joshi V, et al. 2015. Convergent evolution of the genomes of marine mammals. Nat Genet. 47:272–275.

Katoh K, Standley DM. 2013. MAFFT multiple sequence alignment software version 7: improvements in performance and usability. Mol Biol Evol 30:772–780.

Koressaar T, Remm M. 2007. Enhancements and modifications of primer design program Primer3. Bioinformatics. 23:1289-1291.

Lex A, Gehlenborg N, Strobelt H, Vuillemot R, Pfister H. 2014. UpSet: visualization of intersecting sets. IEEE Trans Comput Graph. 20:1983–1992.

Li H, Durbin R. 2009. Fast and accurate short read alignment with Burrows-Wheeler transform. Bioinformatics 25:1754–1760.

Li H, Durbin R. 2011. Inference of human population history from individual whole-genome sequences. Nature 475:493–496.

Li H. 2011. A statistical framework for SNP calling, mutation discovery, association mapping and population genetical parameter estimation from sequencing data. Bioinformatics 27:2987–2993.

Marco-Sola S, Sammeth M, Guigo R, Ribeca P. 2012. The GEM mapper: fast, accurate and versatile alignment by filtration. Nat Methods 9:1185–1188.

O'Leary NA, Wright MW, Brister JR, Ciufo S, Haddad D, McVeigh R, Rajput B, Robbertse B, Smith-White B, Ako-Adjei D, et al. 2016. Reference sequence (RefSeq) database at NCBI: current status, taxonomic expansion, and functional annotation. Nucleic Acids Res. 44:D733–D745.

Sanderson MJ. 2002. Estimating absolute rates of molecular evolution and divergence times: a penalized likelihood approach. Mol Biol Evol. 19:101–109.

Sanderson MJ. 2003. r8s: inferring absolute rates of molecular evolution and divergence times in the absence of a molecular clock. Bioinformatics 19:301–302.

Taylor BL, Chivers SJ, Larese J, Perrin WF. 2007. Generation length and percent mature estimates for IUCN assessments of cetaceans. Administrative Report LJ 07-01. National Marine Fisheries Service, Southwest Fisheries Science Centre.

Untergasser A, Cutcutache I, Koressaar T, Ye J, Faircloth BC, Remm M, Rozen SG. Primer3--new capabilities and interfaces. Nucleic Acids Res. 15:e115.

Warren WC, Kuderna L, Alexander A, Catchen J, Pérez-Silva JG, López-Otín C, Quesada V, Minx P, Tomlinson C, Montague MJ, et al. 2017. The novel evolution of the sperm whale genome. Genome Biol Evol 9:3260–3264.

Werle, E., Schneider, C., Renner, M., Völker, M., and Fiehn, W. (1994) Convenient single-step, one tube purification of PCR products for direct sequencing. Nucleic Acids Res 22 (20): 4354 – 4355

Yandell M, Ence D. 2012. A beginner's guide to eukaryotic genome annotation. Nat Rev Genet. 13:329–342.

Yang Z, Nielsen R. 2002. Codon-substitution models for detecting molecular adaptation at individual sites along specific lineages. Mol Biol Evol. 19:908–917.

Zerbino DR, Achuthan P, Akanni W, Amode MR, Barrell D, Bhai J, Billis K, Cummins C, Gall A, Girón CG, et al. 2018. Ensembl 2018. Nucleic Acids Res. 46:D754–D761.
